# Phosphorylation-dependent regulation of the NOTCH1 intracellular domain by dual-specificity tyrosine-regulated kinase 2

**DOI:** 10.1007/s00018-019-03309-9

**Published:** 2019-10-11

**Authors:** Rosario Morrugares, Alejandro Correa-Sáez, Rita Moreno, Martín Garrido-Rodríguez, Eduardo Muñoz, Laureano de la Vega, Marco A. Calzado

**Affiliations:** 1 Instituto Maimónides de Investigación Biomédica de Córdoba (IMIBIC), Avda. Menéndez Pidal s/n. 14004, Córdoba, Spain; 2 Departamento de Biología Celular, Fisiología e Inmunología, Universidad de Córdoba, Córdoba, Spain; 3 Hospital Universitario Reina Sofía, Córdoba, Spain; 4 Division of Cancer Research, School of Medicine, Jacqui Wood Cancer Centre, James Arrott Drive, Ninewells Hospital and Medical School, University of Dundee, Dundee, Scotland UK; 5 Innohealth Group, Madrid, Spain

**Keywords:** DYRK2, NOTCH1, Degradation, Kinase, Phosphorylation, Cancer

## Abstract

NOTCH proteins constitute a receptor family with a widely conserved role in cell cycle, growing and development regulation. NOTCH1, the best characterised member of this family, regulates the expression of key genes in cell growth and angiogenesis, playing an essential role in cancer development. These observations provide a relevant rationale to propose the inhibition of the intracellular domain of NOTCH1 (Notch1-IC) as a strategy for treating various types of cancer. Notch1-IC stability is mainly controlled by post-translational modifications. FBXW7 ubiquitin E3 ligase-mediated degradation is considered one of the most relevant, being the previous phosphorylation at Thr-2512 residue required. In the present study, we describe for the first time a new regulation mechanism of the NOTCH1 signalling pathway mediated by DYRK2. We demonstrate that DYRK2 phosphorylates Notch1-IC in response to chemotherapeutic agents and facilitates its proteasomal degradation by FBXW7 ubiquitin ligase through a Thr-2512 phosphorylation-dependent mechanism. We show that DYRK2 regulation by chemotherapeutic agents has a relevant effect on the viability, motility and invasion capacity of cancer cells expressing NOTCH1. In summary, we reveal a novel mechanism of regulation for NOTCH1 which might help us to better understand its role in cancer biology.

**Electronic supplementary material:**

The online version of this article (10.1007/s00018-019-03309-9) contains supplementary material, which is available to authorized users.

## Introduction

NOTCH proteins (NOTCH1-4) constitute a receptor family with a widely conserved role in cell cycle, growing, development regulation and cell fate determination [1]. Most of the canonical Notch ligands are transmembrane proteins with an extracellular domain primarily comprised of multiple EGF (Epidermal Growth Factor) repeated similar structures [2]. The best characterised member of this family is NOTCH1, which regulates the expression of key genes in cell growth and angiogenesis, playing an essential role in cancer development. After ligand coupling, NOTCH1 is activated and the receptor is cleaved by a gamma-secretase, leading to the formation of a peptide sequence that corresponds to the intracellular domain of NOTCH1 (Notch1-IC) [3, 4]. In human cells, once Notch1-IC enters the nucleus, and together with the DNA-binding protein CSL (CBF1—Suppressor of Hairless—LAG1) and the co-activator MAML1 (Mastermind-like transcriptional co-activator 1), it stimulates the transcription of target genes related to processes such as proliferation, angiogenesis, cell survival and migration [5, 6].

Functional studies implicate Notch signalling in essentially all of the hallmarks of cancer, being associated with abnormal expression, high mutation rate and poor survival in several cancers such as lung, breast, gastric or lymphoid cancer [7–11]. This oncogenic function of NOTCH in human cancers is related with its capacity to increase cell growth, centered on the ability to induce the expression of Myc [12, 13] and to enhance PI3K-Akt signalling [14]. Additionally, NOTCH has also been described as a mediator of chemoresistance in various human cancers [15, 16]. These observations have provided a rationale for pharmacologic inhibition of Notch1-IC as a potential strategy for treating various cancers [17].

Another potential way to regulate the NOTCH pathway is modulating the activity or stability of Notch1-IC, which is controlled by protein–protein interactions, endocytosis or post-translational modifications (ubiquitination and phosphorylation) [18, 19]. From among them, NOTCH1 degradation by the ubiquitin E3 ligase FBXW7 is considered to be one of the most relevant [20]. FBXW7 binds directly to Notch1-IC promoting its polyubiquitination and proteasomal degradation, which requires phosphorylation at Thr-2512 residue [21–23]. To date, only homeodomain-interacting protein kinase 2 (HIPK2) and MEKK1 have been described to phosphorylate NOTCH1 at Thr-2512, promoting its subsequent proteasomal degradation [24, 25].

Dual-specificity tyrosine-phosphorylation-regulated kinase 2 (DYRK2) is a Ser/Thr kinase that plays key roles in the regulation of proliferation, cell differentiation and survival [26]. DYRK2 contributes to the regulation of various signalling pathways via the phosphorylation of relevant proteins such as NFAT, p53, c-Jun, c-Myc, eIF2Bε, tau, hPXR, glycogen synthase, CRMP4, 4E-BP1, Snail, katanin and SIAH2 [26–30]. Several studies highlighted the importance of DYRK2 impairing the development and progression of human cancers, such as non-small cell lung cancer, esophageal adenocarcinomas, breast cancer and ovarian serous adenocarcinoma [31–34]. In agreement, DYRK2 knockdown increases cancer cell growth and invasion [29]. Similarly, DYRK2 reduces epithelial–mesenchymal transition (EMT) by degrading SNAIL in ovarian cancer [33]. In response to genotoxic stress, ataxia-telangiectasia mutated (ATM) phosphorylates and stabilises DYRK2, which in turn phosphorylates p53 at Ser46 promoting apoptosis [35, 36].

This study describes for the first time DYRK2 as a new upstream negative regulator of the NOTCH1 signalling pathway. We prove that DYRK2 directly interacts with and phosphorylates Notch1-IC at Thr-2512 facilitating its proteasomal degradation by FBXW7. Moreover, we found that DYRK2 modulation by chemotherapeutic agents has a relevant effect on the viability, motility and invasion capacity of cancer cells expressing NOTCH1. In summary, we reveal DYRK2 as a novel negative regulator of NOTCH1 levels and activity, which represents a new control mechanism of the expression and function of this transcription factor with potential implications in cancer.

## Materials and methods

### Cell culture, transfection and reagents

HEK-293T (wt/DYRK2^−/−^/HIPK2^−/−^), HeLa (wt/DYRK2^−/−^/DYRK1A^−/−^), MDA-MB-468 (wt/DYRK2^−/−^), MDA-MB-231 (wt/DYRK2^−/−^), MOR, MCF7, CHO and A549 cells were maintained in Dulbecco’s Modified Eagle’s Medium (DMEM) supplemented with 10% FBS (Fetal Bovine Serum) and 1% (v/v) penicillin/streptomycin (Sigma-Aldrich, St Louis, MO, USA) at 37 °C in a humidified atmosphere containing 5% CO_2_. H727 cells were maintained in Roswell Park Memorial Institute (RPMI) medium at the same conditions. Cell lines were obtained from ATCC (LGC Standards, Teddington, Middlesex, UK) and were routinely tested to be free of mycoplasma and cross contamination. Cell lines validation was performed by a multiplex PCR with Geneprint10 System (Promega, Madison, WI, USA). MG-132 was from Enzo Life Science (Lausen, Switzerland). Transient transfections were carried out with Roti-Fect (Carl Roth, Karlsruhe, Germany) and harvested between 36 and 48 h after transfection. DNA amounts in each transfection were kept constant after the addition of empty expression vector. DYRK2 and Notch1-IC plasmids were previously described or generated in this lab by standard cloning techniques [30]. Point mutants were produced by conventional point mutagenesis. HeLa control and DYRK1A^−/−^ cells were a gift from Dr. Susana de la Luna (Centre for Genomic Regulation, Barcelona, Spain). DYRK2-analogue-sensitive expression plasmid (GFP-DYRK2-AS) was previously described [37]. Myc-tagged Notch1-IC and 4xCSL vectors were provided by Dr. Hee-Sae Park (Korea Basic Science Institute, Gwang Ju, South Korea). pLentiCRISPr-V2 was a gift from Dr. Feng Zhang (Addgene plasmid # 52961). Flag-tagged Notch2-IC and Notch4-IC vectors were a gift from Dr. Raphael Kopan (Addgene plasmids # 20184 and # 20186). HA-tagged Notch1-IC plasmid was a gift from Dr. Urban Lendahl (Addgene plasmid # 47618). HA-tagged Notch1-IC WT and mutant plasmids were kindly provided by Dr. Aifantis (NYU Langone Medical Center, New York, USA). Flag-FBXW7-ΔFbox was kindly provided by Dr. Rocio Sancho (Centre for Stem Cells & Regenerative Medicine King’s College London, UK). Adriamycin (ADR), harmine, etoposide (ETP) and the rest of the reagents were from Sigma-Aldrich. PP1 analogue II 1NM-PP1 (SC-203214) was obtained from Santa Cruz Biotechnology (Santa Cruz, California, USA). Scramble control oligonucleotide siRNA non-targeting pool (D-001810) and ON-TARGET plus SMARTpool against DYRK2 (L-004730-00) were purchased from Dharmacon (Waltham, MA, USA). DYRK2 human recombinant protein was purchased from Abcam (Cambridge, UK).

### Generation of CRISPR/Cas9-cell lines

The endogenous DYRK2 gene was knocked out by transfecting cells with pLentiCRISPR-v2 (which codes for Cas9 and a puromycin cassette) containing gRNAs against the first exon of the short DYRK2 isoform or a combination of gRNAs against the first and the last exon. For HeLa and MDA-MB-468 DYRK2-KO cells, the gRNA sequence used was GCTTGCCAGTGGTGCCAGAG and for MDA-MB-231 and HEK-293T DYRK2-KO cells, the gRNAs used were N-term sequence GCTTGCCAGTGGTGCCAGAG and C-term sequence GAAGCTGAGCTAGAAGGTGG. Control cells were transfected with the empty pLentiCRISPRV2 vector. After transfection, cells were exposed to 2 μg/ml of puromycin for 2 days followed by a medium exchange. Surviving cells were clonally selected (in the case of control cells were used as pool population) by serial dilution, and positive clones were identified by genomic analysis and western blot.

### Western blotting and antibodies

Soluble fractions were obtained after lysis of cells in NP-40 buffer [20 mM Tris–HCl (pH 7.5), 150 mM NaCl, 1 mM phenylmethylsulfonyl fluoride, 10 mM NaF, 0.5 mM sodium orthovanadate, leupeptine (10 μg/ml), aprotinin (10 μg/ml), 1% (v/v) NP-40, and 10% (v/v) glycerol]. Proteins were diluted and boiled at 95 °C in SDS buffer, resolved on sodium dodecyl sulphate polyacrylamide gels (SDS-PAGE), transferred to PVDF membranes, blocked with non-fat milk in TTBS buffer and incubated with primary antibodies. The washed membranes were incubated with appropriate secondary antibodies coupled to horseradish peroxidase, which were detected by chemiluminescence using Clarity™ Western ECL Substrate (Bio-rad Hercules, California, USA). Antibodies against the FLAG epitope (clone M2, A2220) and anti-β-actin (A5316) were purchased from Sigma Aldrich; anti-ubiquitin (P4D1, 3936S) from Cell Signaling Technology (Danvers, Massachusetts, USA). Anti-Notch1 (ab25374), anti-Hes1 (ab71559) and anti-Hes5 (ab25374) were obtained from Abcam. Anti-HA (clone 3F10), anti-myc (clone 9E10), anti-GFP (11814460001) (Roche Molecular Biochemical) and anti-phosphoserine (AB1603) (Millipore, Burlington, Massachusetts, USA) were from the indicated suppliers. Anti-DYRK2 (H80; sc-66867) and anti-DYRK1A (RR7; sc-100376) antibodies were obtained from Santa Cruz Biotechnology. Secondary horseradish peroxidase-coupled antibodies were purchased from Jackson ImmunoResearch Laboratories (Cambridgeshire, UK). Texas Red goat anti-rabbit IgG antibody (T-6391) was from Thermo Fisher Scientific (Waltham, Massachusetts, USA).

### Immunoprecipitation

Cells were washed in PBS and lysed in IP buffer [50 mM Hepes (pH 7.5), 50 mM NaCl, 1% (v/v) Triton X-100, 2 mM EDTA, 10 mM sodium fluoride, 0.5 mM sodium orthovanadate, 10 μg/ml aprotinin, 10 μg/ml leupeptin, and 1 mM PMSF]. Cell lysates were pre-cleared with protein A/G Sepharose (Santa Cruz) and immunoprecipitation was performed on a rotating wheel upon the addition of 1 μg of the indicated antibodies and 25 μl of protein A/G Sepharose beads. Immunoprecipitated proteins were then washed for five times in IP buffer and eluted in 2 × SDS sample buffer, followed by western blotting.

### Luciferase reporter assays

Cells were collected in PBS and lysed in luciferase assay buffer (25 mM Tris–phosphate pH 7.8, 8 mM MgCl_2_, 1 mM DTT, 1% Triton X-100 and 7% glycerol) during 15 min at room temperature in a horizontal shaker. Luciferase assay was performed using Luciferase Assay Reagent (Promega) according to the manufacturer’s instructions. Luciferase activity was measured using an Autolumat LB 953 (Berthold Technologies GmbH, Bad Wildbad, Germany) and normalised with protein concentration.

### Immunofluorescence

Cells were seeded on glass coverslips and 48 h after transfection fixed with 3.7% of pre-warmed paraformaldehyde/PBS for 10 min, permeabilized with 0.1% Triton X-100/PBS for 15 min, blocked with 3% BSA/PBS and incubated overnight with primary antibodies. After being washed with PBS and incubated for 45 min with the secondary antibody, cells were mounted on glass slides with mounting medium-containing DAPI (Vectashield Burlingame, CA, USA). Fluorescence images were captured using an LSM 5 EXCITER (Carl Zeiss MicroImaging GmbH, Oberkochen, Germany) confocal laser scanning microscope using a 40 ×/1.30 oil objective (EC Plan-Neofluar) and ZEN 2008 software (Carl Zeiss MicroImaging GmbH). To determine fluorescent signal, colocalization between different channels the Coloc_2 module was used. The degree of channel colocalization was analysed by considering the following indexes: thresholded Manders’ coefficients A and B and Pearson’s coefficient. To evaluate the spatial relations between channel intensity, we used the ImageJ tool RGB Profiler to create a profile of fluorescence intensity values across a line drawn on the image.

### mRNA extraction and qPCR

Total RNA was extracted using the High Pure RNA Isolation kit (Roche Diagnostics, Switzerland), reverse transcription performed with the iScript cDNA Synthesis kit (Bio-Rad) and real-time PCR carried out in an iCYCLER detection system (Bio-Rad) with iQTM SYBR Green Supermix (Bio-Rad). Amplification efficiencies were validated and normalised against HPRT, and fold change in gene expression was calculated using the $2^{{ - \Delta \Delta C_{t} }}$ method. Primer sequences are available upon request.

### In vitro phosphorylation

Immunoprecipitated myc-tagged Notch1-IC endogenous protein was incubated with 50 ng of commercial recombinant DYRK2 protein (Millipore, 14-669) in kinase buffer (20 mM Hepes pH 7.5, 10 mM MgCl_2_, 1 mM DTT) with or without ATP (0.1 μM). After 60 min of incubation at 37 °C, reactions were stopped using 1 M glycine pH 2.5 in agitation for 20 min at room temperature and A/G beads (Santa Cruz Biotecnology) were removed by centrifugation. Finally, readjustment of pH levels of the supernatant was performed employing 1 M Tris–HCl pH 7.5.

### Cell viability and flow cytometry analyses

For apoptosis studies, cells were harvested and washed in cold PBS and then resuspended in binding buffer consisting of 10 mM Hepes, 140 mM NaCl and 2.5 mM CaCl_2_ pH 7.4. Cells were stained with Annexin V, Alexa Fluor 488 conjugate (Molecular Probes by Life Technologies, Carlsbad, CA, USA) and propidium iodide. Cell cycle distribution and apoptosis were determined by BD FACSCanto™ flow cytometer (BD Biosciences, San Jose, CA, USA) using BD FACSDiva™ software. For cytotoxicity assay, cells were seeded in a 96-well plate and after 12 h YOYO-1 (Life Technologies) was added to a final concentration of 0.1 μM. Object counting analysis was performed using the cell imaging system IncuCyte HD (Essen BioScience).

### Cell motility assay

Cells were seeded in a 96-well Essen ImageLock plate (Essen BioScience, Ann Arbor, Michigan, USA) 24 h after transfection and grown to confluence. After 12 h, the scratches were made using the 96-pin WoundMaker (Essen BioScience), followed by incubation with 10 ng/ml of mitomycin C. Wound images were taken every 60 min for 24 h and the data analysed by the integrated metric Relative Wound Density part of the live content cell imaging system IncuCyte HD (Essen BioScience).

### Cell invasion assay

Invasion assays were performed in Boyden chamber using a 48-well Neuro Probe, Inc. insert system (Gaithersburg, MD, USA). Polyethylene membrane inserts (8.0 μm pore size) were precoated with 200 μg/μl of Matrigel^®^ Matrix (Corning^®^, Corning, NY, USA) (in coating buffer 0.01 M Tris and 0.7% NaCl). Cells were subcultured in an mw6 plate, and 24 h prior the assay, FBS was removed from the media and ADR was added in the specific conditions. Then, cells were seeded with 2.5 × 10^4^ cells per insert (cells suspended in 50 μl in DMEM, in addition to 25 μl FBS free DMEM in the bottom side of the chamber) and incubated at 37 °C, 5% CO_2_ for 12 h. Then, the membrane was washed at least three times for 10 min with PBS. The membranes were then cut out of the inserts by a scalpel, dyed in methyl violet for 30 min and mounted between two thin cover slips. The total number of migrated cells was counted for each group (*n* = 4) with an inverted microscope. Only cells which had completely migrated through the membrane were counted.

### Enrichment of His-tagged proteins

Cells were collected in PBS and pellets resuspended in lysis buffer (6 M guanidinium-HCl, 0.1 M Na_2_HPO_4_/NaH_2_PO_4_, 0.01 M Tris–HCl [pH 8], 5 mM imidazole and 0.01 M β-mercaptoethanol). Samples were sonicated and cell debris was removed by centrifugation. Supernatants were mixed with 75 μl of equilibrated Ni–NTA resin (Quiagen, Hilden, Germany), followed by incubation for 4 h at room temperature on a rotating wheel. Precipitates were washed once with lysis buffers, once in wash buffer (8 M urea, 0.1 M Na_2_HPO_4_/NaH_2_PO_4_, 0.01 M Tris–HCl [pH 6.8], 5 mM imidazole, and 0.01 M β-mercaptoethanol), and twice in wash buffer plus 0.1% Triton X-100. Proteins were eluted in 75 μl of 0.2 M imidazole, 0.15 M Tris–HCl (pH 6.8), 30% glycerol, 0.72 M β-mercaptoethanol and 5% SDS for 20 min at room temperature with gentle agitation and further analysed by immunoblotting.

### Data analysis

Protein abundance in tumor tissue was obtained from The Human Protein Atlas database as antibody staining level (not detected, low, medium and high) per patient [38]. Data were accessed via the R hpar package. Gene alteration frequencies were calculated using the TCGA PanCancer dataset that includes 10967 samples across 33 different tumor types [39]. To calculate the alteration frequencies, the number of samples containing a missense/non-sense mutation or a deep deletion for a given gene was divided by the total number of samples in a given cancer type. Data were accessed via the cBioPortal web service using the R cgdsr package [40]. Images were evaluated and quantified using the Image J (http://rsbweb.nih.gov/ij/). Data are expressed as mean ± SD. Differences were analysed by Student’s *t* test. *P* < 0.05 was considered significant. Statistical analysis was performed using GraphPad Prism^®^ version 6.01 (GraphPad, San Diego, CA, USA).

## Results

### Notch1-IC protein levels are modulated by DYRK2

**Fig. 1:**
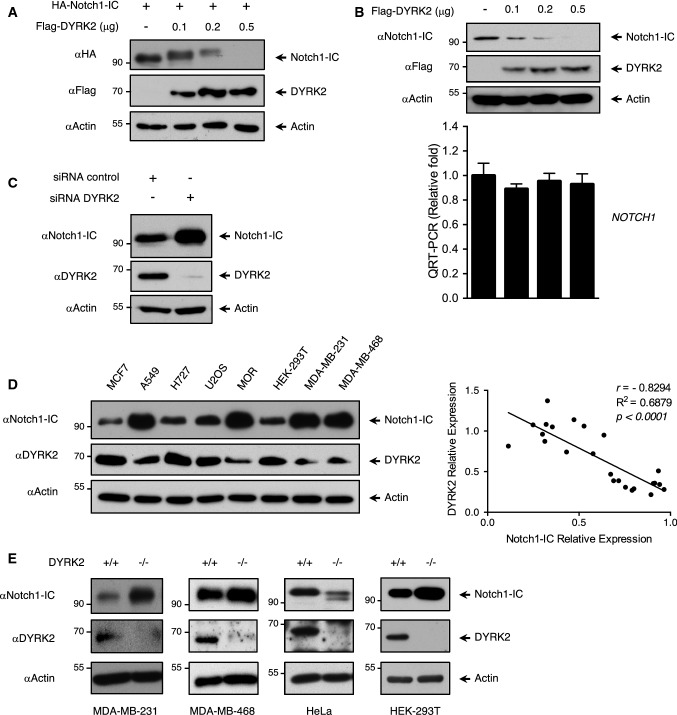
NOTCH1 protein levels are modulated by DYRK2. **a** HEK-293T cells were transfected (2 × 10^5^ cells in a 35-mm dish, increasing amounts of DYRK2) with the indicated plasmids and lysed 48 h after transfection. Protein expression was evaluated by immunoblotting. We show a representative blot of three independent experiments. **b** HEK-293T cells (2 × 10^5^ cells in a 35 mm dish) were transfected with the indicated amounts of DYRK2, harvested and lysed. One fraction was used to analyse endogenous Notch1-IC protein levels, while another aliquot was used to analyse Notch1-IC mRNA levels by quantitative PCR. Data are mean ± SD of *n* = 3. We show a representative blot of three independent experiments. **c** HEK-293T cells were transfected with DYRK2 or scrambled (control) siRNAs, lysed after 4 days of culture and Notch1-IC or DYRK2 analysed by western blot. We show a representative blot of three independent experiments. **d** Notch1-IC and DYRK2 endogenous protein levels were analysed in the indicated cell lines by immunoblotting. We show a representative blot of three independent experiments (left panel). Notch1-IC and DYRK2 signals from three independent experiments were quantified, normalised to actin protein levels and correlation was analysed (right panel). **e** Endogenous protein levels of DYRK2 and Notch1-IC were evaluated in MDA-MB-231, MDA-MB-468, HeLa and HEK-293T both WT and DYRK2^−/−^ by immunoblot. We show a representative blot of three independent experiments

To identify new potential DYRK2 interaction partners, we performed an immunoprecipitation assay followed by mass spectrometry (Fig. S1a and Supplementary Materials and methods). As NOTCH1 and other members of the family showed positive results, we decided to focus on this protein in detail. We first co-expressed Notch1-IC alone or with increasing amounts of DYRK2 in HEK-293T cells. Expression of DYRK2 resulted in a dose-dependent decrease in Notch1-IC protein levels, which was accompanied by the appearance of slower migrating bands (Fig. 1a). The activity of the rest of the members of human DYRK subfamily protein kinases (DYRK1A, DYRK1B, DYRK2, DYRK3, and DYRK4) was also analysed. As shown in Supplementary Figure S1b, DYRK1A and DYRK1B overexpression showed a similar effect as compared to DYRK2, which was not observed with DYRK3 and 4. Similarly, DYRK2 overexpression resulted in a decrease of the rest of NOTCH human family members (NOTCH2, NOTCH3 and NOTCH4) (Fig. S1c).

Next, we decided to analyse the impact of DYRK2 expression on the levels of endogenous Notch1-IC. Transfection of increasing amounts of DYRK2 led to a dose-dependent decrease of endogenous Notch1-IC protein levels without affecting its mRNA expression (Fig. 1b). Similar results were obtained with DYRK1B (Fig. S1d). It has been previously described that DYRK1A phosphorylates NOTCH1 [41]. In this sense, the specificity of the antibodies and plasmids used for DYRK2 detection was analysed (Fig. S1e). In addition, DYRK2 effect on endogenous Notch1-IC protein levels was reanalysed in DYRK1A knockout cells obtaining similar results (Fig. S1f). Then we analysed the effect of knocking down DYRK2 using a specific siRNA. In agreement with our previous results, DYRK2 depletion increased Notch1-IC levels (Fig. 1c) as well as its half-life (Fig. S1g), further proving that Notch1-IC basal levels were regulated by DYRK2.

Our results suggested that DYRK2 might negatively regulate NOTCH1 protein levels, and thus we hypothesised that the endogenous levels of DYRK2 and NOTCH1 might show a correlation. To test our hypothesis, we analysed the levels of these two proteins in eight different cell lines, and as shown in Fig. 1d, a negative correlation was observed. Next, to further confirm the ability of this kinase to regulate Notch1-IC, DYRK2 knockout cell lines were generated by CRISPR/Cas9 gene-editing tools and Notch1-IC protein levels were evaluated. As shown in Fig. 1e, specific stable DYRK2 knockout resulted in increased levels of Notch1-IC in three of the four cell lines tested. In the case of HeLa, DYRK2 knockout led to the appearance of faster migrating bands, which might reflect unphosphorylated NOTCH1. Collectively, these results demonstrate that DYRK2 negatively regulates Notch1-IC levels.

### DYRK2 phosphorylates Notch1-IC in vivo and in vitro

**Fig. 2:**
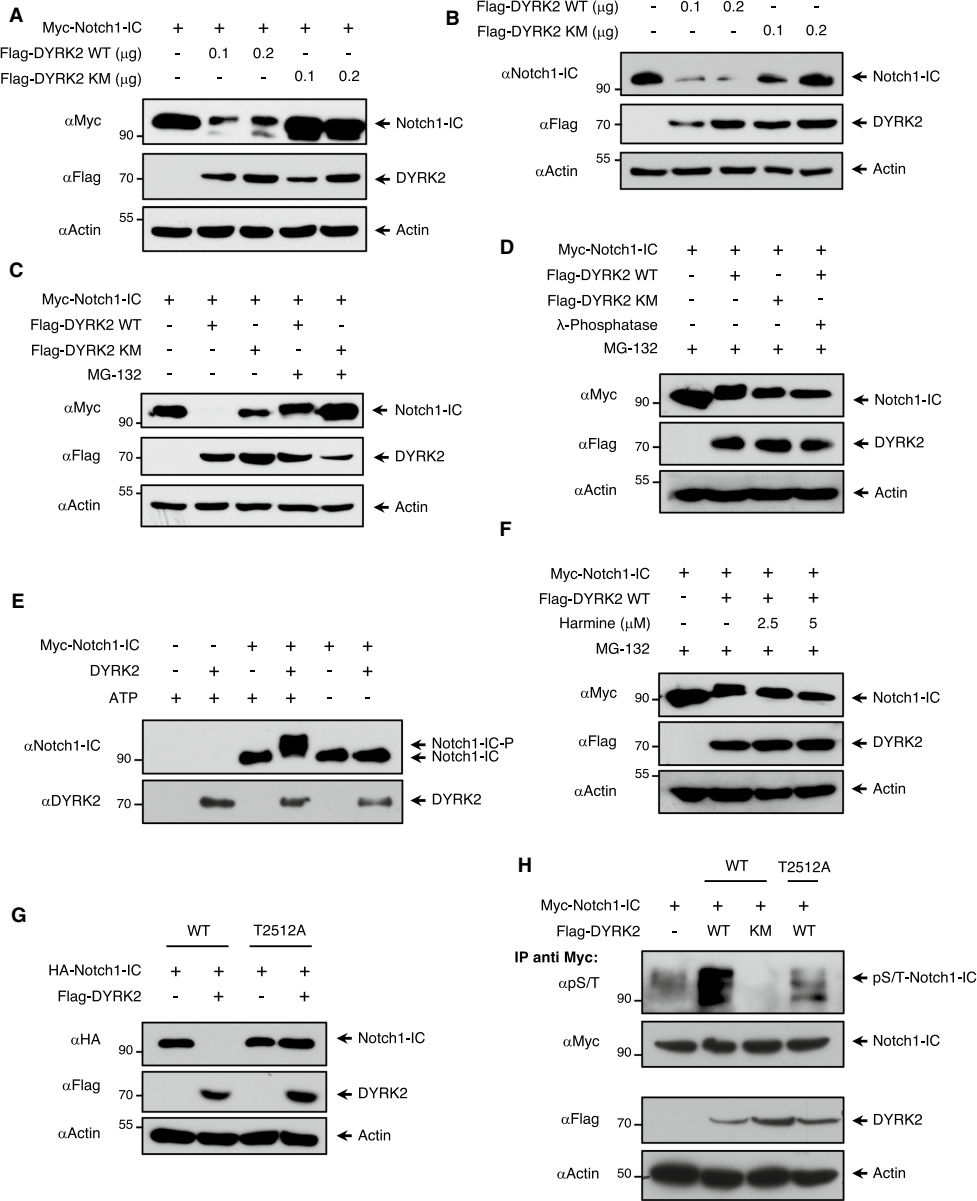
DYRK2 phosphorylates Notch1-IC. **a** HEK-293T cells were transfected to express Myc-Notch1-IC and increasing amounts of Flag-DYRK2 wild type (WT) or kinase mutant (KM). Cell lysates were analysed by immunoblotting with the indicated antibodies. We show a representative blot of four independent experiments. **b** HEK-293T cells were transfected to express DYRK2 WT or KM. Twenty-four hours post-transfection, cells were lysed and protein expression was analysed by immunoblot with the indicated antibodies. We show a representative blot of three independent experiments. **c** HEK-239T cells were co-transfected with the indicated plasmids and then treated or not for 12 h with the proteasome inhibitor MG-132 (10 μM). Cell lysates were analysed by immunoblotting with anti-Myc and Flag antibodies. We show a representative blot of three independent experiments. **d** HEK-293T cells were transfected with the indicated plasmids and treated with MG-132 for 12 h and were lysed in phosphatase inhibitor-free buffer in the absence or presence of *λ*-phosphatase. Electrophoretic mobility was determined by immunoblotting. We show a representative blot of four independent experiments. **e** Immunoprecipitated Notch1-IC endogenous protein from HEK-293T cells was incubated with DYRK2 recombinant protein in the presence or absence of ATP (0.1 μM). Electrophoretic mobility was determined by immunoblotting with the indicated antibodies. We show a representative blot of four independent experiments. **f** HEK-239T cells were co-transfected with the indicated plasmids and after 36 h treated with MG-132 in the presence or not of harmine for 12 h before lysis. Protein expression was analysed by immunoblot with the indicated antibodies. We show a representative blot of three independent experiments. **g** Cells were transfected to express HA-Notch1-IC WT or HA-Notch1-IC T2512A (threonine 2512 mutated to alanine) in the presence or not of Flag-DYRK2 WT. Cells were further cultivated and lysed and protein expression was analysed by immunoblot with the indicated antibodies. We show a representative blot of three independent experiments. **h** HEK-293T cells were transfected to express Myc-Notch1-IC WT or Myc-Notch1-IC T2512A in the presence or not of Flag-DYRK2-WT or KM and, after 36 h, treated with MG-132 for 8 h and lysed. A fraction was subjected to immunoprecipitation (IP) using anti-Myc antibody. After elution phosphorylation was revealed with an anti-phospho-serine/threonine antibody, while exogenous Notch1-IC protein levels were visualised with an anti-Myc antibody by western blotting (top panel). The remaining extract fraction was tested for the occurrence of the indicated proteins (lower panel). We show a representative blot of three independent experiments

Based on the capacity of DYRK2 to induce the appearance of slower migrating bands compatible with phospho-forms of Notch1-IC, next we analysed whether DYRK2 kinase activity was necessary for its effect on Notch1-IC levels. We co-expressed Notch1-IC with either increasing amounts of DYRK2 or a kinase point mutant version (DYRK2 KM). As shown in Fig. 2a, DYRK2 overexpression led to a reduction of Notch1-IC levels concomitant with the appearance of upshifted bands. By contrast, Notch1-IC levels were not altered in the presence of the DYRK2 KM. Similar results were obtained at the endogenous level (Fig. 2b). To test whether that reduction in protein levels was mediated by protein degradation, we decided to examine this effect in the presence or absence of the proteasome inhibitor MG-132. As shown in Fig. 2c, the addition of MG-132 considerably prevented DYRK2-mediated Notch1-IC degradation and stabilised band mobility. To confirm that the upshifted Notch1-IC bands were phosphorylated forms, we incubated cell extracts with λ-phosphatase in the presence of MG-132. As shown in Fig. 2d, λ-phosphatase treatment transformed a slower electrophoretic band mobility into a faster migrating movement, similar to those obtained in response to DYRK2 KM expression. To evaluate the ability of DYRK2 to directly phosphorylate Notch1-IC, we performed an in vitro kinase assay (Fig. 2e). The presence of recombinant DYRK2 showed the occurrence of upper Notch1-IC bands, which appeared only in the presence of ATP. Moreover, the relevance of the DYRK2 kinase activity for Notch1-IC was highlighted by experiments with chemical inhibitors. As shown in Figure S2a, treatment with the pan-specific DYRK inhibitor harmine inhibited the negative effect of DYRK2 on Notch1-IC levels, and also caused a clear increase in the motility of the Notch1-IC bands in the presence of MG-132 (Fig. 2f). Similar results were obtained with curcumin, another DYRK2 inhibitor [42] (Fig. S2b). Altogether, these experiments demonstrate that DYRK2 directly phosphorylates Notch1-IC.

To identify the Notch1-IC sites phosphorylated by DYRK2, we analysed different relevant residues mutated to alanine involved in Notch1-IC regulation previously described [22]. Co-expression of the mutants with DYRK2 and subsequent analysis of their electrophoretic behaviour showed that mutation of Thr-2512 significantly reduced DYRK2-mediated Notch1-IC degradation (Fig. S2c). Similar results were obtained when we compared the co-expression of DYRK2 with Notch1-IC WT vs T2512A (Fig. 2g), indicating that phosphorylation of Thr-2512 is necessary for the effect of DYRK2 on Notch1-IC stability. To further prove that DYRK2 phosphorylates Notch1-IC in cells, NOTCH1 WT and T2512A phospho-deficient mutant constructs were transfected together with DYRK2 WT or DYRK2 KM, immunoprecipitated, and their phosphorylation status was analysed with a phospho-serine/threonine antibody (as there is no specific phospho-T2512 NOTCH1 antibody available) (Fig. 2h). Mutation of Thr-2512 to alanine clearly reduced the phosphorylation of Notch1-IC mediated by DYRK2 (by comparing lanes 2 and 4). However, DYRK2 was still able to induce some phosphorylation in the T2512A Notch1-IC mutant, indicating that it might not be the only site phosphorylated by DYRK2 in cells. Collectively, these results clearly demonstrate that DYRK2-mediated phosphorylation of Notch1-IC at threonine 2512 is crucial for its degradation.

### DYRK2 regulates proteasomal degradation of Notch1-IC

**Fig. 3:**
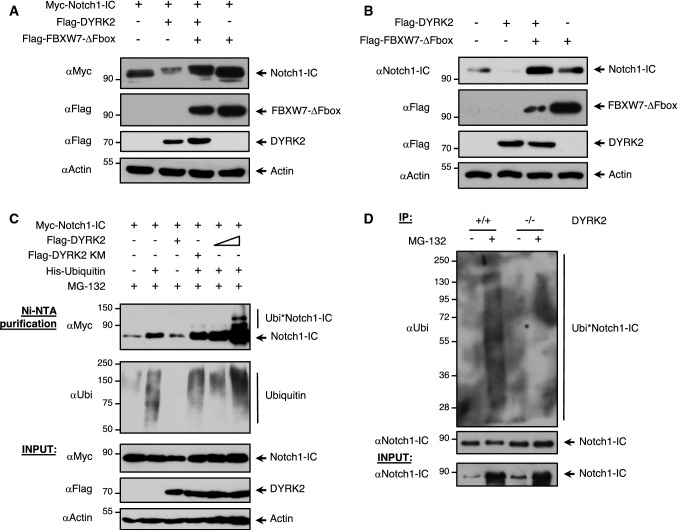
DYRK2 regulates Notch1-IC protein levels via Fbxw7-mediated proteasomal degradation. **a** HEK-293T cells were co-transfected to express Notch1-IC together with DYRK2, and the levels were evaluated in response to FBXW7 dominant negative (FBXW7-∆Fbox) lacking the F-box domain that recruits ubiquitination machinery. We show a representative blot of three independent experiments. **b** HEK-293T cells were transfected with Flag-DYRK2 in the presence or absence of a dominant-negative form of FBXW7. Endogenous Notch1-IC protein levels were evaluated by western blotting. We show a representative blot of three independent experiments. **c** HEK-293T cells were transfected with expression plasmids encoding Flag-tagged DYRK2, Myc-tagged Notch1-IC and His-tagged ubiquitin. After 36 h, cells were incubated in the presence of MG-132 (10 μM) during 12 h and lysed under denaturing conditions. His-tagged ubiquitin was purified with Ni–NTA agarose columns and ubiquitinated Notch1-IC was analysed by western blotting. A fraction was tested for the occurrence of the indicated proteins (INPUT). We show a representative blot of three independent experiments. **d** Wild-type and DYRK2^−/−^MDA-MB-231 cells were stimulated or not with MG-132 during 9 h, lysed and subjected to immunoprecipitation using anti-Notch1-IC antibody. A small fraction of the lysate was tested for the occurrence of Notch1-IC (INPUT). The precipitates were subjected to western blot analysis with anti-Notch1-IC or anti-Ubi antibodies. We show a representative blot of three independent experiments

As shown in Fig. 2c, treatment with the proteasome inhibitor MG-132 restored the level of Notch1-IC upon DYRK2 expression (lanes 2 and 4). These results indicated that DYRK2 decreased the stability of Notch1-IC through a ubiquitin/proteasome-dependent process. Based on the previous reports showing that Thr-2512 phosphorylation facilitated Notch1-IC proteasomal degradation through FBXW7 [21], we decided to evaluate if this ubiquitin ligase was implicated in the degradation of Notch1-IC mediated by DYRK2. To analyse the role of FBXW7 in this process, we first co-expressed DYRK2 and Notch1-IC in the presence or absence of a dominant-negative form of FBXW7 lacking the F-box (FBXW7ΔFbox). As shown in Fig. 3a, FBXW7ΔFbox expression recovered the level of Notch1-IC decreased by DYRK2, preserving the reduction of the band mobility. Similar results were obtained at the endogenous level (Fig. 3b). These results showed that FBXW7 is important for the degradation of NOTCH1 mediated by DYRK2.

We next examined the effect of DYRK2 on Notch1-IC ubiquitination. We co-expressed Myc-Notch1-IC and His-Ubiquitin with or without different concentrations of DYRK2 and DYRK2 KM in the presence of MG-132 and analysed the ubiquitination status of Notch1-IC. As shown in Fig. 3c, Notch1-IC polyubiquitination became more evident in the presence of increasing concentrations of DYRK2. Furthermore, we examined the effect of DYRK2 depletion on the basal level of Notch1-IC polyubiquitination, comparing control and DYRK2^−/−^ cells in the presence or absence of MG-132. As shown in Fig. 3d, ubiquitination levels of Notch1-IC were significantly lower in cells lacking DYRK2. Collectively, these data show that DYRK2 promotes basal Notch1-IC polyubiquitination and proteasomal degradation via FBXW7.

### DYRK2 interacts and colocalizes with Notch1-IC

**Fig. 4:**
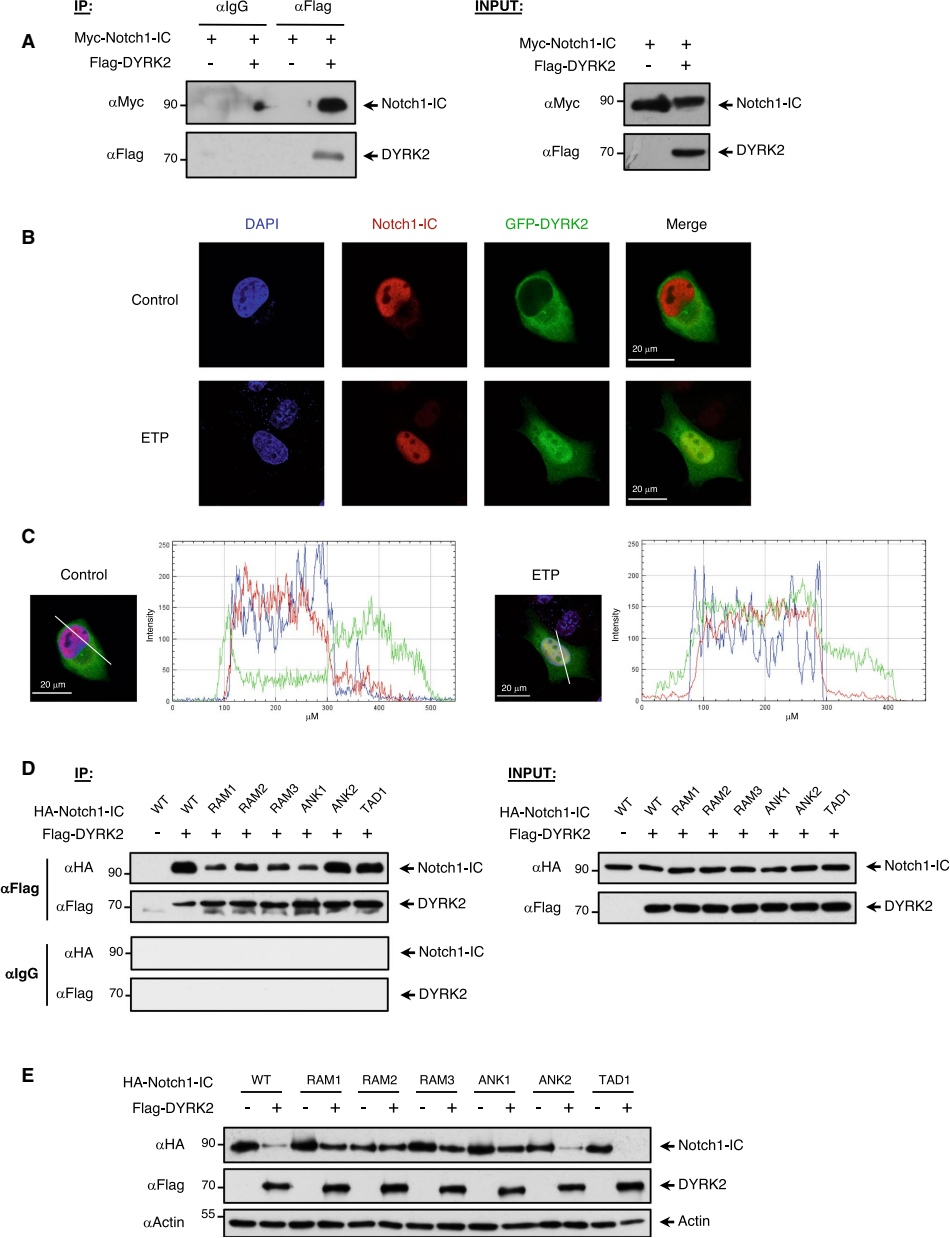
Notch1-IC interacts and colocalizes with DYRK2. **a** HEK-293T cells were transfected with expression plasmids Myc-tagged Notch1-IC and Flag-DYRK2 as indicated and after 36 h the proteasome inhibitor MG-132 was added for another 12 h to avoid Notch1 degradation. Cells were lysed and subjected to immunoprecipitation (IP) using anti-Flag antibody. After elution, Myc-Notch1-IC protein was detected by western blotting. A small fraction (5%) of the lysate was tested for the occurrence of the indicated proteins by immunoblot (INPUT). The positions and molecular weights (in kDa) are indicated. We show a representative blot of three independent experiments. **b** CHO cells were transfected with GFP-DYRK2 and analysed for the subcellular localization of DYRK2 and endogenous Notch1-IC proteins by confocal microscopy stimulated or not during 6 h with ETP (10 μM). Nuclear DNA was stained with DAPI. We show a representative picture where overlapping localization in merged pictures is shown in yellow. **c** Fluorescence intensity profiles through the white line shown indicate GFP-DYRK2 and Notch1-IC cellular localization in both control and DNA damage conditions. Pearson’s coefficient (0.65) and thresholded Manders’ coefficients *A* and *B* (*A* = 0.789; *B* = 0.773) were calculated for both situations. **d** HEK-293T cells were transfected with Flag-DYRK2 and HA-Notch1-IC plasmids (WT and mutant versions) as indicated, and after 36 h the proteasome inhibitor MG-132 (10 μM) was added for another 12 h. Cells were lysed, subjected to immunoprecipitation (IP) using anti-Flag antibody and the different proteins detected by western blotting (left panel). A small fraction (5%) of the lysate was tested by immunoblot for the occurrence of the indicated proteins (INPUT, right panel). We show a representative blot of three independent experiments. **e** HEK-293T cells were co-transfected with HA-Notch1-IC or the indicated mutants either alone or along with DYRK2. After 36 h, cells were lysed and the stability of Notch1-IC was revealed by immunoblotting. We show a representative blot of three independent experiments

Next, we analysed the ability of DYRK2 to interact with Notch1-IC. We first co-expressed Myc-Notch1-IC alone or in the presence of Flag-DYRK2 and performed coimmunoprecipitation assays. As shown in Fig. 4a, DYRK2 coimmunoprecipitated efficiently with Notch1-IC. Then, we analysed the subcellular localization of both proteins and the effect of DNA damage. Confocal microscopy showed that GFP-DYRK2 and endogenous Notch1-IC mainly colocalize in the nucleus, with a high degree of nuclear localization of DYRK2 in cells stimulated with ETP (Pearson’s coefficient = 0.65 and Manders’ coefficients of *A* = 0.789; *B* = 0.773) (Fig. 4b and c).

To map the interaction sites of Notch1-IC with DYRK2, first we performed an in vitro interaction peptide array experiment. A peptide library consisting of overlapping fragments representing the entire Notch1-IC or DYRK2 proteins was incubated with GST-DYRK2 or GST-Notch1-IC, respectively, using GST as a control. Detection of bound material by antibodies showed six potential binding regions of Notch1-IC with DYRK2. Likewise, DYRK2 showed two potential binding regions with Notch1-IC, which correspond to the C-terminal region of the protein (Fig. S4a and S4b). Next, to validate the functional relevance of the regions present in Notch1-IC, all were mutated and tested for their interaction with DYRK2 (Notch1-IC mutant constructs. Fig. S4c). As shown in Fig. 4d, the individual mutation of the regions in the RAM domain and one adjacent in the ANK (ankyrin) domain caused a marked reduction in the ability to coimmunoprecipitated efficiently with DYRK2. Furthermore, the ability of DYRK2 to negatively regulate Notch1-IC protein levels was strongly reduced (Fig. 4e and S4d). All these results clearly prove the direct interaction between Notch1-IC and DYRK2, and suggest the existence of more than one region responsible for binding in both proteins, highlighting the possible relevant role of the RAM domain present in Notch1-IC.

### Genotoxic stress induces Notch1-IC degradation mediated by DYRK2

**Fig. 5:**
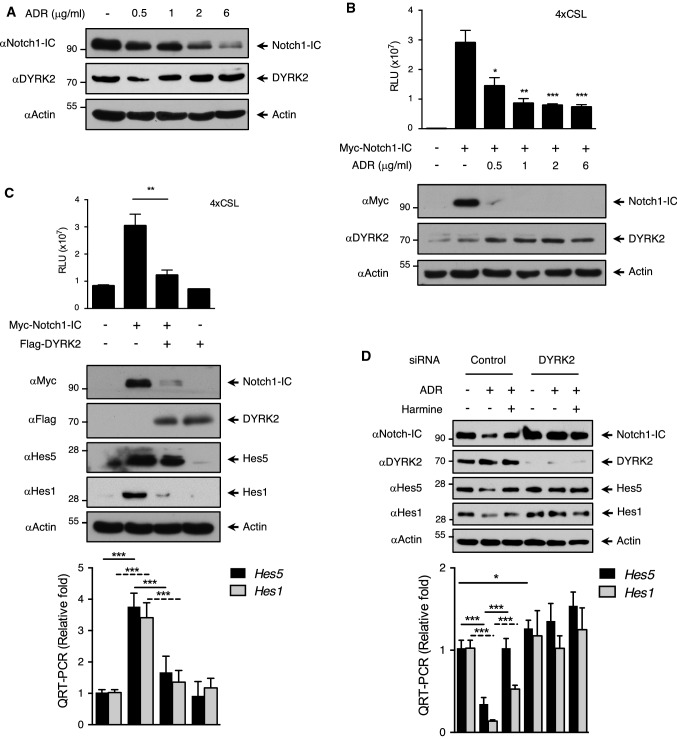
Genotoxic stress affects Notch1-IC signalling via DYRK2. **a** HEK-293T cells were stimulated with increasing concentrations of ADR for 12 h, lysed and endogenous levels of Notch1-IC and DYRK2 measured by immunoblotting. We show a representative blot of four independent experiments. **b** HEK-293T cells were transfected with the indicated plasmids and the 4xCSL-luciferase reporter and 24 h later stimulated with the indicated doses of ADR for another 12 h. Cells were lysed and one aliquot was used for the luciferase reporter assay (upper panel), while another fraction was used to analyse the levels of the indicated protein by immunoblot. We show a representative blot of three independent experiments. Data are mean ± SD of *n* = 3 experiments. *P < 0.05, **P < 0.01, ***P < 0.001. **c** HEK-293T cells were transfected with the indicated plasmids. One aliquot was used for the luciferase reporter assay and immunoblot (upper panel), while another was used to analyse *Hes5* and *Hes1* mRNA levels by qPCR (lower panel). We show a representative blot of three independent experiments. Data are mean ± SD of n = 3 experiments. ***P < 0.001. **d** MDA-MB-231 cells were transfected with DYRK2 or scrambled (control) siRNAs and, after 3 days of culture, stimulated or not with ADR (2 μg/ml) for 12 h in the presence or absence of harmine (5 μM). One fraction was used to analyse the levels of the indicated protein by immunoblot (upper panel), while another was used to analyse Hes5 and Hes1 mRNA levels by qPCR (lower panel). We show a representative blot of three independent experiments. Data are mean ± SD of *n* = 3 experiments. *P < 0.05, ***P < 0.001

Next, we decided to evaluate the physiological relevance of the observed effect of DYRK2 on Notch1-IC levels. Among the stimuli able to regulate the activity of this kinase, the response to DNA damage upon exposure to genotoxic stress [30, 35, 36] stands out. Therefore, we tested the ability of DNA damage to regulate NOTCH1 signalling via DYRK2. HEK-293T cells were stimulated with increasing concentrations of DNA-damaging agent adriamycin (ADR) and protein levels of both Notch1-IC and DYRK2 were evaluated by western blot. As depicted in Fig. 5a, upregulation of DYRK2 in response to ADR was inversely correlated with Notch1-IC protein levels. Similar results were obtained in MDA-MB-231 cells (Fig. S5a), DYRK1A knockout cells (Fig. S5b) and with other genotoxic agents such as etoposide and cisplatin in different cell lines (Fig. S5c and S5d). Next, we evaluated the consequences of genotoxic stress on Notch1-IC transcriptional activity. In agreement with our previous results, ADR treatment impaired Notch1-IC transcriptional activity (Fig. 5b). Similar results were obtained with HIPK2 knockout cells (Fig. S5e) and after DYRK2 overexpression (Fig. S5f). We then investigated whether modulation of DYRK2 levels affected the transcriptional activity of Notch1-IC. As shown in Fig. 5c, ectopic expression of DYRK2 reduced Notch1-IC transcriptional activity. Moreover, Hes5 and Hes1 induction by Notch1-IC at both mRNA and protein levels was reduced by DYRK2 overexpression. Similarly, to demonstrate the role of DYRK2 kinase activity on the control of Notch1-IC transcriptional activity, we used an analogue-sensitive DYRK2 form (DYRK2-AS), which presents a mutation in the gatekeeper residue and is selectively sensitive to PP1 inhibitors [43]. As shown in Figure S5g, the specific inhibition of DYRK2 activity by PP1 analogue II stimulation reduced drastically the effect of DYRK2 on Notch1-IC protein levels with clear effects on Hes5 at both mRNA and protein levels. Taken together, these results further suggest that DYRK2 modulates Notch1-IC regulation in response to genotoxic stress.

To demonstrate the role of DYRK2 on the regulation of Notch1-IC in response to DNA damage, we assessed the effect of DYRK2 knockdown with siRNA. As shown in Fig. 5d, DYRK2 depletion avoided adriamycin-mediated reduction of Notch1-IC (lanes 2 and 5). In the same sense, Hes5 and Hes1 expression (RNA and protein) reduced by ADR treatment was restored by harmine and DYRK2 knockdown (lanes 2, 3 and 5) in MDA-MB-231 cells. Similar results were obtained in HEK-293T cells (Fig. S5h). Finally, we compared the effect of Notch1-IC ectopic expression in knockout cells lacking DYRK2. As shown in Figure S5i, Notch1-IC-induced expression of luciferase reporter gene was higher in DYRK2 knockout cells than in control cells. Altogether these data suggest that DYRK2 has a relevant role on Notch1-IC protein levels and activity in response to DNA damage.

### DYRK2 modulation modifies Notch1-IC physiological effects

**Fig. 6:**
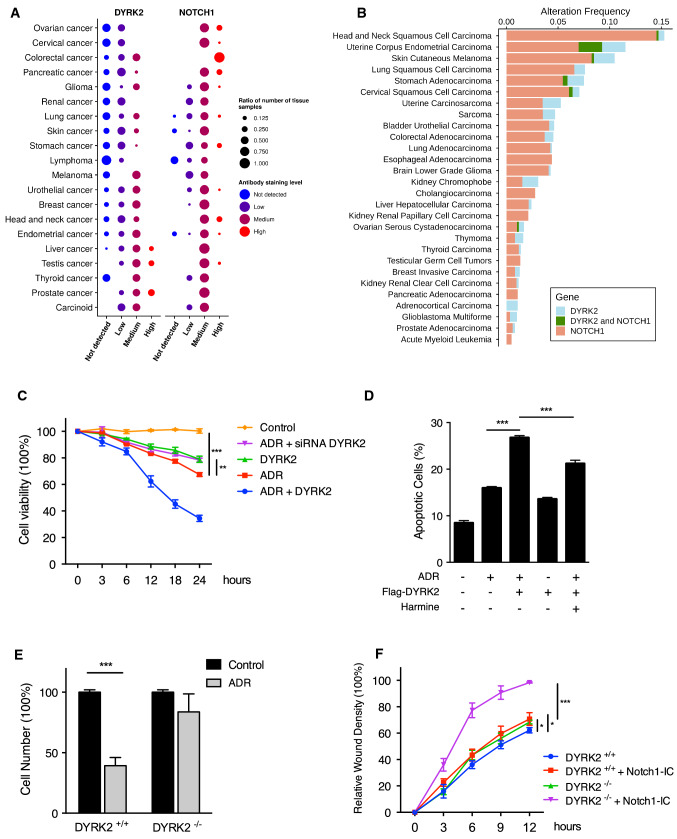
DYRK2 inhibition increases Notch1-IC tumorigenesis effect in breast cancer. **a** DYRK2 and NOTCH1 protein abundance in tumour tissues obtained from The Human Protein Atlas. Column and circle colour show the antibody stain level observed in tumour tissues. The point size indicates the number of patients showing particular expression to the total patients. The tumour tissues were sorted based on the abundance score differences between proteins. To calculate this, every staining level was assigned to a number (Not detected: 1, Low: 2, Medium: 3 and High: 4) and multiplied by the number of patients for each tissue and protein. Then, the absolute mean differences were calculated for every tumour tissue. **b** DYRK2 and NOTCH1 mutation frequency separately or together (missense, non-sense or deep deletions) for every tumour type included in the TCGA PanCancer dataset. **c** MDA-MB-231 cells were transfected with DYRK2 or scrambled (control) siRNAs or Flag-DYRK2 as indicated, and after 48 h of culture stimulated or not with ADR (2 μg/ml). Cell viability was determined using YOYO-1 fluorescence. Data are mean ± SD of n = 3 experiments. **P < 0.01, ***P < 0.001. **d** MDA-MB-231 cells were transfected or not with Flag-DYRK2 and after 36 h of culture stimulated or not with ADR (2 μg/ml) for 12 h in the presence or absence of harmine (5 μM) and used for apoptosis analysis by Annexin V/PI staining. Cell viability was measured by flow cytometry. Data are mean ± SD of *n* = 3 experiments. **P < 0.01, ***P < 0.001. **e** MDA-MB-231 WT and DYRK2 ^−/−^ cells were stimulated or not with ADR (2 μg/ml) for 12 h and used for matrigel motility assays. Data are mean ± SD of *n* = 3 experiments. ***P < 0.001. **f** MDA-MB-231 WT and DYRK2 ^−/−^ cells were transfected or not with Flag-Notch1-IC and after 36 h used for cell motility assays. Data are mean ± SD of *n* = 3 experiments. *P < 0.05, ***P < 0.001

Finally, in order to investigate the clinical significance of our findings, we first analysed data from the public database The Human Protein Atlas to determine the protein abundance of DYRK2 and NOTCH1 in tumour tissues. As we previously observed in different cell lines, a considerable number of tissues present in a high number of patients showed low levels of DYRK2 expression and a high NOTCH1 abundance, from which the differences observed in ovarian, cervical, colorectal or pancreatic cancer stand out (Fig. 6a). Similarly, the analysis of the frequency of loss-of-function mutations on DYRK2 and/or NOTCH1 in tumours showed that mutations on DYRK2 and NOTCH1 occur very rarely together, suggesting that both proteins might be in the same pathway (Fig. 6b). Next, we examined the effect of DYRK2 modulation on cell viability and apoptosis in response to ADR. We observed that knocking down DYRK2 in MDA-MB-231 cells increased cell viability in response to ADR (Fig. 6c). However, the opposite effect was observed with cells overexpressing DYRK2, showing a strong reduction on cell survival in response to ADR. In the same context, the percentage of apoptotic cells upon exposure to ADR was increased by DYRK2 overexpression, and a significant reduction was observed in the presence of harmine in MDA-MB-231 (Fig. 6d) and MDA-MB-468 cells (Fig. S6a). Similarly, DYRK2 overexpression affected the expression of genes involved in cell viability such as *BCL2* (Fig. S6b). Additionally, DYRK2 is necessary for adriamycin-induced suppression of cell invasion (Fig. 6e). Finally, cell motility experiments in MDA-MB-231 (Fig. 6f) and MDA-MB-468 cells (Fig. S6c) showed that, although in the presence of DYRK2 the protein levels of Notch1-IC affected cancer cell migration significantly, Notch1-IC overexpression considerably increased cell migration of DYRK2-KO cells, suggesting that DYRK2 restrains Notch1-mediated cancer cell migration. Associated with these results, changes in the expression of genes related with mobility and invasion, such as *FGF*, *TFG*-*β, TNF* or *OCT*-*4*, were observed (Fig. S6d). Altogether, these results indicate a new role of DYRK2 in cancer cell migration/invasion through the regulation of Notch1-IC levels.

## Discussion

In the present work, we describe DYRK2 as a new kinase that regulates NOTCH1 pathway via phosphorylation, controlling its protein levels and activity in response to DNA damage. Different reports have shown how some kinases have the ability to regulate Notch1-IC by phosphorylation, thus facilitating its subsequent ubiquitination. Phosphorylation of the PEST domain is a substrate for recognition by FBXW7, which binds directly to Notch1-IC promoting its polyubiquitination and proteasomal degradation recruiting the components of an SCF ubiquitin ligase complex degradation [18, 20, 21]. Although different kinases such as cyclin C and various CDKs (CDK3, CDK8 and CDK19) [44] have been described able to regulate Notch1-IC by phosphorylation of the PEST domain, requirement of previous Thr-2512 phosphorylation for FBXW7 interaction has been described in bibliography [22, 23]. To date, to our knowledge, only MEKK1 and HIPK2 have been described to be able to phosphorylate Thr-2512 and promote proteasomal degradation of Notch1-IC by this pathway [18, 25]. Our findings show that DYRK2 is also able to directly phosphorylate Notch1-IC at Thr-2512 in the PEST domain and facilitate its proteasomal degradation.

Our results related to the interaction between Notch1-IC and DYRK2 suggest the possible relevant role of the RAM domain present in Notch1-IC. Previous studies suggested that the primary function of the RAM region is to recruit Notch1-IC to CSL [45, 46], which, together with MAML1, stimulate the transcription of target genes. In this sense, further studies should be done to clarify whether the interaction with DYRK2 in response to some stimuli could presumably inhibit Notch1-IC downstream signalling through interaction with this domain.

It is also important to mention the evolutive proximity between DYRK2 and HIPK2. Both kinases belong to the CMGC group and are evolutionarily very close [26]. They are both ubiquitinated by MDM2 [36, 47] and SIAH2 ubiquitin ligases, and present both common and specific substrates for each of them [30, 48]. Similarly, although both respond to certain common stimuli, they may also be present in some pathways exclusively. In the specific case of cellular response to DNA damage stimulus, both kinases are able to phosphorylate p53 at Ser46 to irreversibly induce p53-dependent apoptosis [35, 49]. Our findings indicate that Notch1-IC regulation seems to be also common for both kinases, being the action mechanism of DYRK2 described in this study HIPK2 independent (Figure S5e and data not shown). The concerted regulation in response to DNA damage of Notch1-IC executed by DYRK2 and also HIPK2 may represent a fail-safe mechanism to ensure a corrected reduction of Notch1-IC levels in this context. However, further studies should be done to elucidate the connection between these two pathways. Similarly, analysing the response of Notch1-IC to other stimuli able to regulate the activity or expression of DYRK2, such as hypoxia [30], serum starvation [50], β-adrenergic stimulation [51], LPS [52] or heat shock [37], would be of interest.

In the context of chemotherapy resistance, one of the most important problems in cancer treatment, understanding the molecular mechanisms implicated in the DNA damage response (DDR) pathway is crucial. In this sense, it has been reported that overexpression of Notch1-IC in lung and liver cancer cells increases resistance to cisplatin [53, 54]. Similarly, NOTCH1 plays a direct negative regulatory role on DDR following ionising radiation treatment by interacting with ATM and disrupting its activation [55]. On the other hand, Li et al. [56], recently described that cisplatin induces expression of Notch1-IC in a dose-dependent manner in cervical cancer cells. In fact, in some specific types of tumours such as skin cancer, small cell lung cancer or hepatocellular carcinoma, contradictory data indicate that NOTCH1 signalling could be playing anti-proliferative rather than oncogenic roles [57]. In this study, we provide new insights into the consequences of exposure to DNA damage on the NOTCH1 signalling pathway, since different chemotherapeutic agents (adriamycin, etoposide or cisplatin) promote Notch1-IC inhibition mediated by DYRK2 in different cell lines.

Previous studies have broadly shown that perturbation of the NOTCH1 signalling pathway is linked to the pathogenesis of important lung diseases, in particular, lung cancer and lung lesions [58, 59]. However, it has also been proved to play a key role in breast cancer [60, 61] and prostate cancer [62]. Additionally, Notch1-IC aberrant overexpression correlates with leukaemia [63] and breast cancer [9, 64]. Although DYRK2 distinct role in cancer development has been broadly proved, there is controversy concerning its pro- or anti-tumour potentials. However, various studies have shown that DYRK2 is down-regulated in various cancer tissues such as lung, breast, prostate and colon, associated with poor patient prognosis [32, 50, 65–69]. The data showing the correlation in the levels of both proteins in tumour tissue (Fig. 6a) agree with our in vitro data and suggest that the degradation of Notch1-IC by DYRK2 might also be relevant in cancer patients. Additionally, our results prove that DYRK2 Thr-2512 direct phosphorylation is an important milestone in Notch1-IC regulation. These results might clarify previous analyses focused on the relevance of Thr-2515 mutation in some cancers such as human T cell acute lymphoblastic leukaemia [70].

**Fig. 7:**
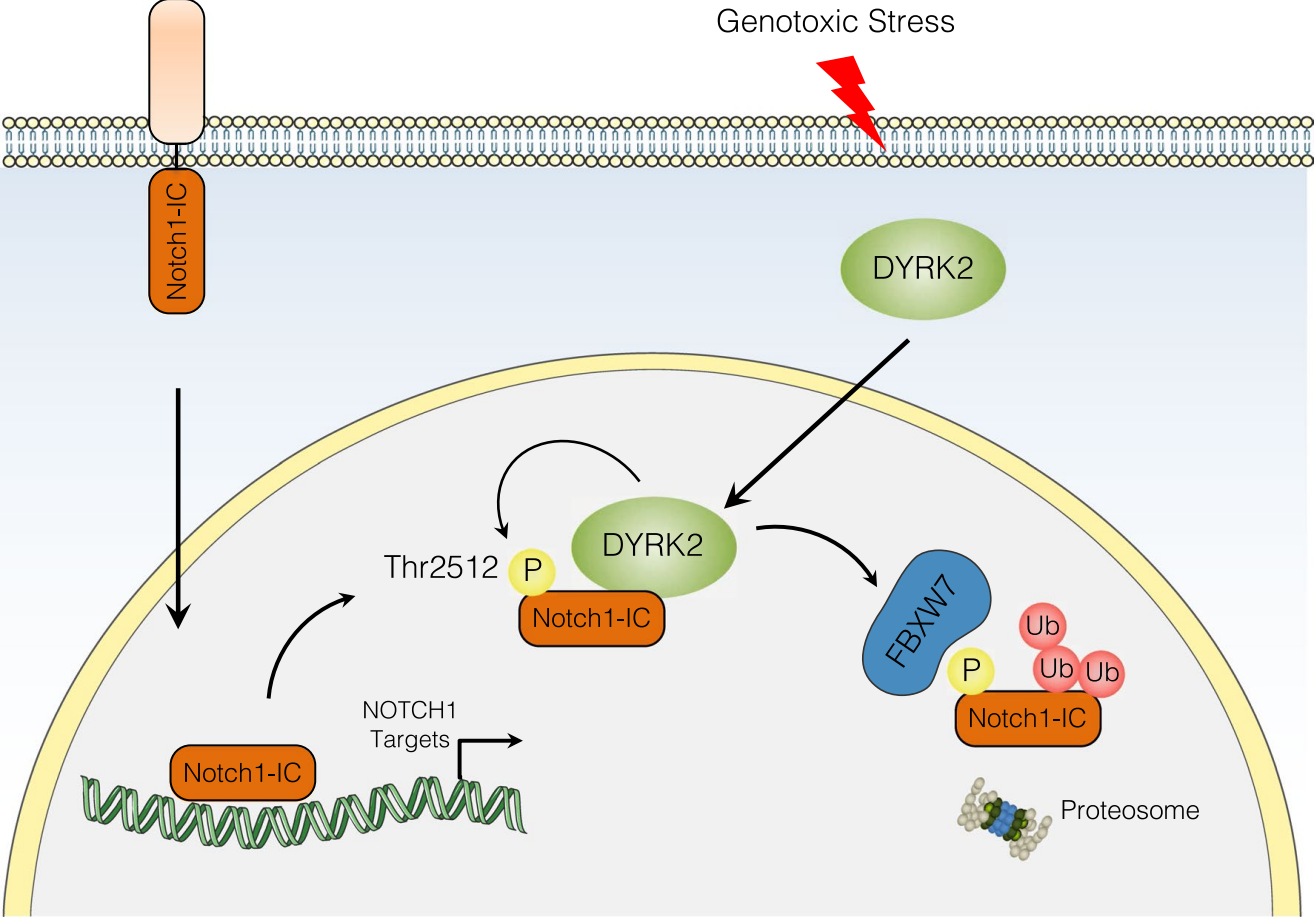
Schematic model for the ability of DYRK2 to regulate Notch1-IC. Under genotoxic stress, DYRK2 is able to regulate Notch1-IC by FBXW7-dependent proteasome degradation by phosphorylating residue Thr-2512

In summary, our results show the ability of DYRK2 to regulate Notch1-IC stability affecting its transcriptional activity. In response to DNA damage, DYRK2 phosphorylated Notch1-IC and facilitated its proteasomal degradation by FBXW7 (Fig. 7). We propose that this new regulatory mechanism induced by chemotherapeutic agents has an influence on cancer cells behaviour. Further studies are needed to understand the potential implications for those tumours with over-expressed Notch1-IC.

### Electronic supplementary material

Below is the link to the electronic supplementary material.

Supplementary material 1 (DOCX 31 kb)

Supplementary material 2 (PDF 9864 kb)
