# Supplementary material for: Phosphorylation-dependent regulation of the NOTCH1 intracellular domain by dual-specificity tyrosine-regulated kinase 2
Source: Cell Mol Life Sci. 2019 Oct 11;77(13):2621–39. doi: 10.1007/s00018-019-03309-9 (PMC7320039; doi:10.1007/s00018-019-03309-9)
Supplement: Supplementary file 1 — Supplementary material 1 (DOCX 31 kb) [file 18_2019_3309_MOESM1_ESM.docx]

**Supplementary Material for:**

**Phosphorylation-dependent regulation of the NOTCH1 intracellular domain by dual-specificity tyrosine-regulated kinase 2.**

Rosario Morrugares^1,2,3*^, Alejandro Correa-Sáez^1,2,3*^, Rita Moreno^4^, Martín Garrido-Rodríguez^1,2,3,5^, Eduardo Muñoz^1,2,3^, Laureano de la Vega^4^ and Marco A. Calzado^1,2,3^

^1^ Instituto Maimónides de Investigación Biomédica de Córdoba (IMIBIC), Córdoba, Spain.

^2^ Departamento de Biología Celular, Fisiología e Inmunología, Universidad de Córdoba, Córdoba, Spain.

^3^ Hospital Universitario Reina Sofía, Córdoba, Spain.

^4^ Division of Cancer Research, School of Medicine, Jacqui Wood Cancer Centre, James Arrott Drive, Ninewells Hospital and Medical School, University of Dundee, Dundee, Scotland.

^5^ Innohealth Group, Madrid, Spain

* These two authors contributed equally to this work.

**Corresponding Author:** Marco A. Calzado Ph.D.

Instituto Maimónides de Investigación Biomédica de Córdoba (IMIBIC)

Avda. Menendez Pidal s/n. 14004, Córdoba, Spain

Phone: + 34 957213762

e-mail: [mcalzado@uco.es](mailto:mcalzado@uco.es)

**Supplementary figures.**

**Supplementary Figure S1. a** HEK-293T cells were washed in PBS, lysed and endogenous DYRK2 immunoprecipitation (IP) performed with anti-DYRK2 antibody or rabbit IgG. After elution, DYRK2 protein was detected by western blotting. A small fraction (5%) of the lysate was tested for the occurrence of the indicated proteins by immunoblot (INPUT). Experiments were performed and analysed in triplicate (Upper panel). Summary table that shows the results obtained for DYRK2 and NOTCH family from the immunoprecipitation assay coupled to mass spectrometry, including number of peptides identified after IP with anti-DYRK2 antibody or rabbit IgG in each assay (see Supplementary Material and Methods). Spectrums of some of the peptides identified for DYRK2, NOTCH1, NOTCH2 and NOTCH3. **b** HEK-293T cells were co-transfected with the indicated plasmids and after 36 h were lysed and protein expression was evaluated by immunoblot with the indicated antibodies. We show a representative blot of three independent experiments. **c** HEK-293T cells were co-transfected with expression vectors encoding Flag-DYRK2 and the different NOTCH family members. After 36 h protein levels were analysed by immunoblotting with the indicated antibodies. We show a representative blot of three independent experiments. **d** HEK-293T cells were transfected to express HA-DYRK1A, HA-DYRK1B, HA-DYRK2, HA-DYRK3 and HA-DYRK4. Cells were further cultivated, lysed and protein expression was analysed by immunoblot with the indicated antibodies. Notch1-IC mRNA levels were evaluated by quantitative PCR. Data are mean ± SD of n = 3. We show a representative blot of three independent experiments. **e** HEK-293T cells were transfected with the indicated plasmids and after 36 h lysed and protein expression was evaluated by immunoblot with the indicated antibodies. We show a representative blot of three independent experiments. **f** HeLa WT and DYRK1A^-/-^ cells (2x10^5^ cells in a 35 mm dish) were transfected with the indicated amounts of DYRK2 and lysed 36 h later. Protein expression was analysed by immunoblot with the indicated antibodies. We show a representative blot of three independent experiments. **g** HEK-293T cells were transfected with DYRK2 or scrambled (control) siRNAs, after 3 days treated with the protein synthesis inhibitor cycloheximide (CHX) (50 μg/ml) for 0.5, 1, 2, 4, and 6 h, lysed and Notch1-IC analysed by western blot using actin expression as the loading control. The graph represents the mean ± SD of band density from 3 different experiments.

**Supplementary Figure S2. a** HEK-293T cells were co-transfected with the indicated plasmids and after 36 h incubated with 5 μM of harmine for 12 h before lysis. Cell extracts were subjected to western blot and revealed with the indicated antibodies. We show a representative blot of four independent experiments. **b** HEK-293T cells were co-transfected with the indicated plasmids and after 36 h incubated with harmine (5 μM) or curcumine (5 μM) for 6 h before lysis. Cell extracts were subjected to western blot and revealed with the indicated antibodies. We show a representative blot of three independent experiments. **c** HEK-293T cells were transfected to express HA-Notch1-IC WT or the indicated mutants (threonine mutated to alanine) in the presence or not of Flag-DYRK2-WT. Cells were further cultivated and lysed and protein expression was analysed by immunoblot with the indicated antibodies. We show a representative blot of three independent experiments.

**Supplementary Figure S4. a** Notch1-IC and DYRK2 representations with the regions responsible for interaction. Recombinant GST-DYRK2 or GST-Notch1-IC proteins (or GST control protein) were incubated with a peptide array library covering the complete sequence of Notch1-IC or DYRK2 respectively, and bound proteins were revealed by immunoblot. We show a representative blot of two independent experiments. **b** Peptide library of Notch1-IC and DYRK2 was used to localize direct interactions using recombinant proteins GST-DYRK2 and GST-Notch1-IC. Notch1-IC and DYRK2 peptides required for the interaction determined by peptide array screening are shown in the figure. **c** Schematic representation of WT and mutant constructs of Notch1-IC. To alter the protein conformation in the regions identified by peptide array screening previously, amino acids in the centre were mutated to alanine (A). In the case of HA-Notch1-RAM3, alanine and valine (amino acids with hydrophobic side chain) were mutated to lysine (K) and glutamic acid (E) (amino acids with electrically charged side chains) respectively. **d** Notch1-IC signals from three independent experiments from Figure 4E were quantified and normalized to actin protein levels using the Image J. Results are expressed as % Inhibition of Notch1-IC signal WT and of each mutant in response to DYRK2. **P* < 0.05, ***P* < 0.01.

**Supplementary Figure S5. a** MDA-MB-231 or **b** HeLa DYRK1A^-/-^ cells were stimulated with increasing concentrations of ADR for 12 h, lysed and endogenous levels of Notch1-IC and DYRK2 measured by immunoblotting. We show a representative blot of three independent experiments. **c** H727 cells were stimulated with increasing concentrations of etoposide for 12 h, lysed and endogenous levels of Notch1-IC and DYRK2 measured by immunoblotting. We show a representative blot of three independent experiments. **d** MDA-MB-231 cells were stimulated with the indicated concentrations of cis-platin for 12 h and then lysed to examine Notch1-IC and DYRK2 protein levels by western blot using the indicated antibodies. We show a representative blot of three independent experiments. **e** HEK-293T HIPK2^-/-^ cells were transfected with the indicated plasmids and the 4xCSL-luciferase reporter and 24 h later stimulated with the indicated doses of ADR for another 12 h. Cells were lysed and the luciferase activity was measured. We show a representative blot of three independent experiments. Data are mean ± SD of n = 3 experiments. **P* < 0.05, ***P* < 0.01. **f** HEK-293T cells were transfected with the indicated plasmids and 36 h later lysed. One aliquot was used for the luciferase reporter assay (upper panel), while another aliquot was used to analyse the levels of the indicated protein by immunoblot. We show a representative blot of three independent experiments. Data are mean ± SD of n = 3 experiments. ****P* < 0.001. **g** HEK-293T cells were transfected with the indicated plasmids and after 48 hours treated for a further 3 hours with PP1 analog II 1-NM-PP1 (3 μM). One aliquot was used for the immunoblot (upper panel), while another was used to analyse Hes5 mRNA levels by qPCR (lower panel). We show a representative blot of three independent experiments. Data are mean ± SD of n = 3 experiments. ***P* < 0.01, ****P* < 0.001. **h** HEK-293T cells were transfected with DYRK2 or scrambled (control) siRNAs and after 3 days of culture stimulated or not with ADR (2 μg/ml) for 12 h in the presence or absence of harmine (5 μM). One fraction was used to analyse the levels of the indicated protein by immunoblot (upper panel), while another was used to analyse *Hes5* and *Hes1* mRNA levels by qPCR (lower panel). We show a representative blot of three independent experiments. Data are mean ± SD of n = 3 experiments. **P* < 0.05, ****P* < 0.001. **i** MDA-MB-231 WT and DYRK2 ^-/-^ cells were co-transfected with myc-Notch1-IC and 4xCS L-luciferase reporter plasmids and 36 h later lysed. One aliquot was used for the luciferase reporter assay (upper panel), while another aliquot was used to analyse the levels of the indicated protein by immunoblot. We show a representative blot of three independent experiments. Data are mean ± SD of n = 3 experiments. ***P* < 0.01.

**Supplementary Figure S6. a** MDA-MB-468 cells were transfected or not with Flag-DYRK2 and after 36 hours of culture stimulated or not with ADR (2 μg/ml) for 12 h in the presence or absence of harmine (5 μM), and used for apoptosis analysis by Annexin V/PI staining. Cell viability was measured by flow cytometry. Data are mean ± SD of n = 3 experiments. ****P* < 0.001. **b** MDA-MB-231 cells were transfected or not with Flag-DYRK2 and after 36 hours of culture stimulated or not with ADR (2 μg/ml) for 12 h. Cells were lysed and *BCL2* mRNA levels analysed by quantitative PCR. Data are mean ± SD of n = 3. **c** MDA-MB-468 WT and DYRK2^-/-^ cells were transfected or not with Flag-Notch1-IC and after 36 h used for cell motility assays. Data are mean ± SD of n = 3 experiments. **P* < 0.05, ****P* < 0.001. **d** MDA-MB-231 WT and DYRK2^-/-^ cells were transfected or not with Flag-Notch1-IC and after 36 hours used to analyse the mRNA levels of the indicated genes by quantitative PCR. Data are mean ± SD of n = 3. ***P* < 0.01, ****P* < 0.001.

**Supplementary Material and Methods**

1. **Immunoprecipitation coupled to mass spectrometry**
   1. *Immunoprecipitation*

HEK-293T cells were washed in PBS and lysed in IP buffer [50 mM Hepes (pH 7.5), 50 mM NaCl, 1% (v/v) Triton X-100, 2 mM EDTA, 10 mM sodium fluoride, 0.5 mM sodium orthovanadate, 10 μg/ml aprotinin, 10 μg/ml leupeptin, and 1 mM PMSF]. Cell lysates were pre-cleared with protein A/G Sepharose (Santa Cruz) and immunoprecipitation was performed on a rotating wheel upon the addition of 1 μg of anti-DYRK2 or IgG control. Immunoprecipitated proteins were then washed ten times in IP buffer and eluted in elution buffer (0.2 M glycine, pH 2.3/ 0.5% Igepal CA-630). Experiments were performed and analysed in triplicate.

- 1. *Sample preparation for LC-MS analysis*

Lysed cells were cleaned to remove contaminants by protein precipitation with TCA/acetone and solubilized in 50 μl of 0.2% RapiGest (Waters, Milford, MA, USA) in 50 mM ammonium bicarbonate. Total protein was quantified using Qubit Protein Assay Kit (Thermo Fisher Scientific, Waltham, MA, USA) and 50 μg proteins from each sample were digested with trypsin. Briefly, protein samples were incubated with 5 mM DTT at 60 °C for 30 min, and then with 10 mM iodoacetamide at room temperature for 30 min in darkness. Sequencing Grade Modified Trypsin (Promega, Madison, WI, USA) was added (ratio 1:40 trypsin:protein) and samples were incubated at 37 °C for 2 h. Afterwards, trypsin was added again (ratio 1:40) and samples were incubated at 37 °C for 15 h. RapiGest was suppressed by precipitation with 0.5% TFA at 37 °C for 1 h and centrifugation. The final volume was adjusted with milliQ water and ACN to a final concentration of 0.5 μg peptide/μL (2.25% ACN and 0.2% TFA), and 1× of the iRT peptides (Biognosis AG, Schlieren/Zurich, Switzerland) were spiked in each sample.

- 1. *LC-MS analysis*

Peptide solutions were analysed in triplicate by a shotgun approach by nanoLC-MS/MS. Samples (3 μl) were analysed with a nano-LC system Ekspert nLC415 (Eksigent, Dublin, CA, USA) using an Acclaim PepMap RSLC C18 column (75 μm × 25 cm, 3 μm, 100 Å) (Thermo Fisher Scientific) at a flow rate of 300 nl/min. Water and ACN, both containing 0.1% formic acid, were used as solvents A and B, respectively. The gradient run consisted of 5% to 30% B for 120 min. Peptides eluted were directly injected into a hybrid quadrupole-TOF mass spectrometer Triple TOF 5600+ (Sciex, Redwood City, CA, USA) operated with a top 65 data-dependent acquisition system (DDA) using positive ion mode. A NanoSpray III ESI source (Sciex) was used for the interface between nano-LC and MS, applying 2600 V. The acquisition mode consisted of a 250 ms survey MS scan from 350 to 1250 m/z, followed by an MS/MS scan from 230 to 1700 m/z (60 ms acquisition time, 350 mDA mass tolerance, rolling collision energy) of the top 65 precursor ions from the survey scan. The fragmented precursors were then added to a dynamic exclusion list for 15 s, excluding any singly charged ions from the MS/MS analysis. Peptide and protein identifications were performed using Protein Pilot software v5.0 (Sciex) with a human UniProtKB concatenated target-reverse decoy database, specifying cysteines as modification and trypsin as enzyme used for digestion. The false discovery rate (FDR) was set to 1% for peptides and proteins. Those peptide proteins identified in the IgG control samples were subtracted from the later analysis. Protein were scores analysing Total ProtScore, Unused ProtScore, confident peptides and % Cov (95).
