# Supplementary material for: Phosphorylation-dependent regulation of the NOTCH1 intracellular domain by dual-specificity tyrosine-regulated kinase 2
Source: Cell Mol Life Sci. 2019 Oct 11;77(13):2621–39. doi: 10.1007/s00018-019-03309-9 (PMC7320039; doi:10.1007/s00018-019-03309-9)
Supplement: Supplementary file 2 — Supplementary material 2 (PDF 9864 kb) [file 18_2019_3309_MOESM2_ESM.pdf]

A

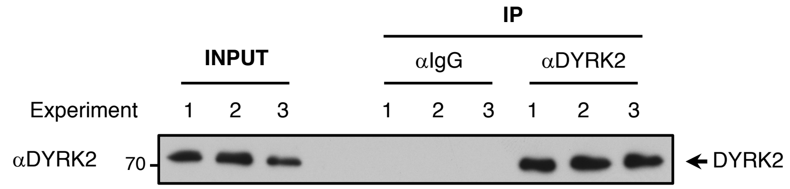


| Representative Accession | Name | Control IGs Peptides | IP DYRK2 Peptides | Unused ProtScore | Total ProtScore | Confident Peptides | %Seq Cov - peptides >95% conf |
|---|---|---|---|---|---|---|---|
| sp\|Q92630\|DYRK2_HUMAN | Dual specificity tyrosine-phosphorylation-regulated kinase 2 OS=Homo sapiens GN=DYRK2 PE=1 SV=3 | 0\|0\|0 | 18\|8\|11 | 28,06 | 28,08 | 18 | 32,4 |
| sp\|P46531\|NOTC1_HUMAN | Neurogenic locus notch homolog protein 1 OS=Homo sapiens GN=NOTCH1 PE=1 SV=4 | 0\|0\|0 | 11\|10\|11 | 14,60 | 25,07 | 12 | 33,1 |
| sp\|Q04721\|NOTC2_HUMAN | Neurogenic locus notch homolog protein 2 OS=Homo sapiens GN=NOTCH2 PE=1 SV=3 | 0\|0\|0 | 22\|44\|19 | 54,31 | 54,31 | 44 | 23,3 |
| sp\|Q9UM47\|NOTC3_HUMAN | Neurogenic locus notch homolog protein 3 OS=Homo sapiens GN=NOTCH3 PE=1 SV=2 | 0\|0\|0 | 13\|8\|9 | 21,95 | 21,99 | 13 | 6,1 |

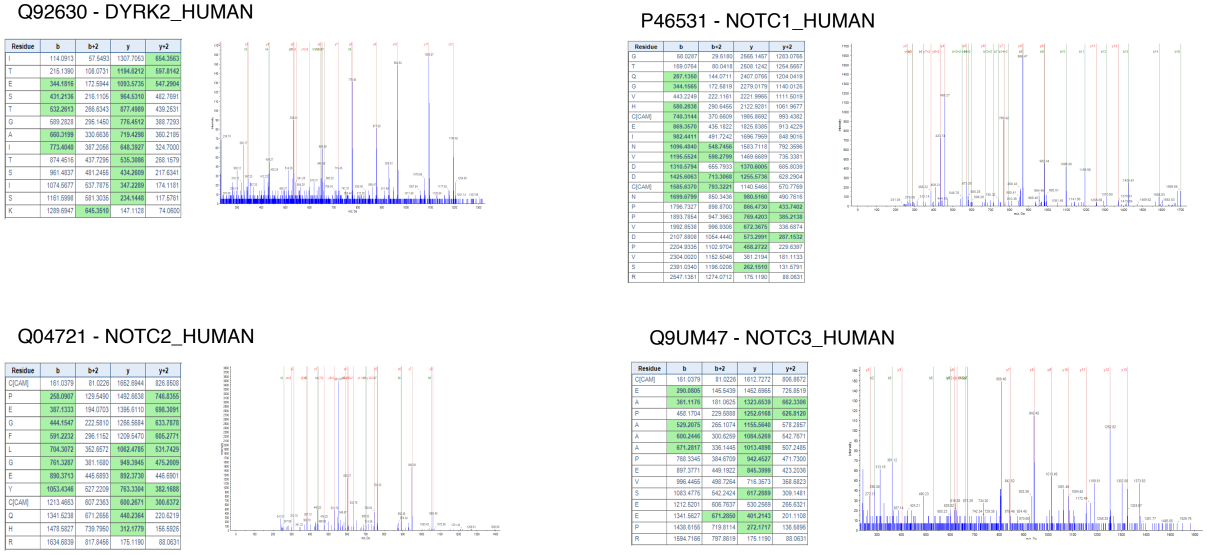


B

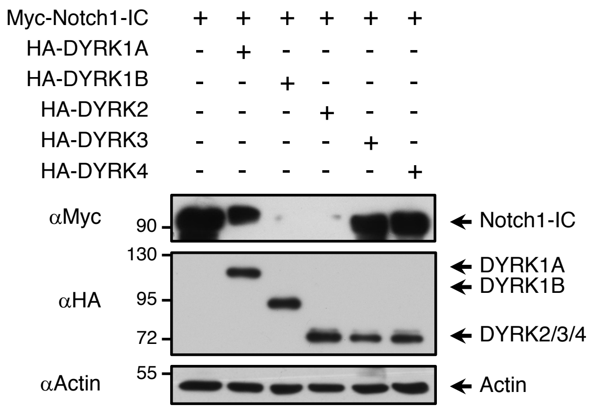


C

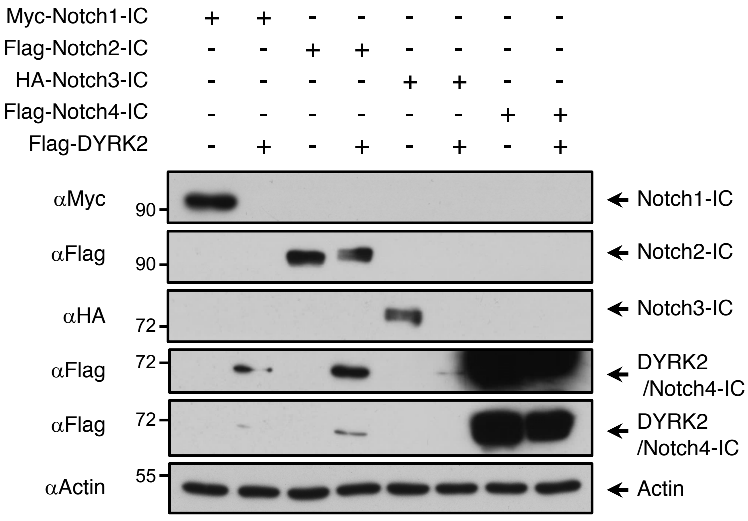


Fig. S1

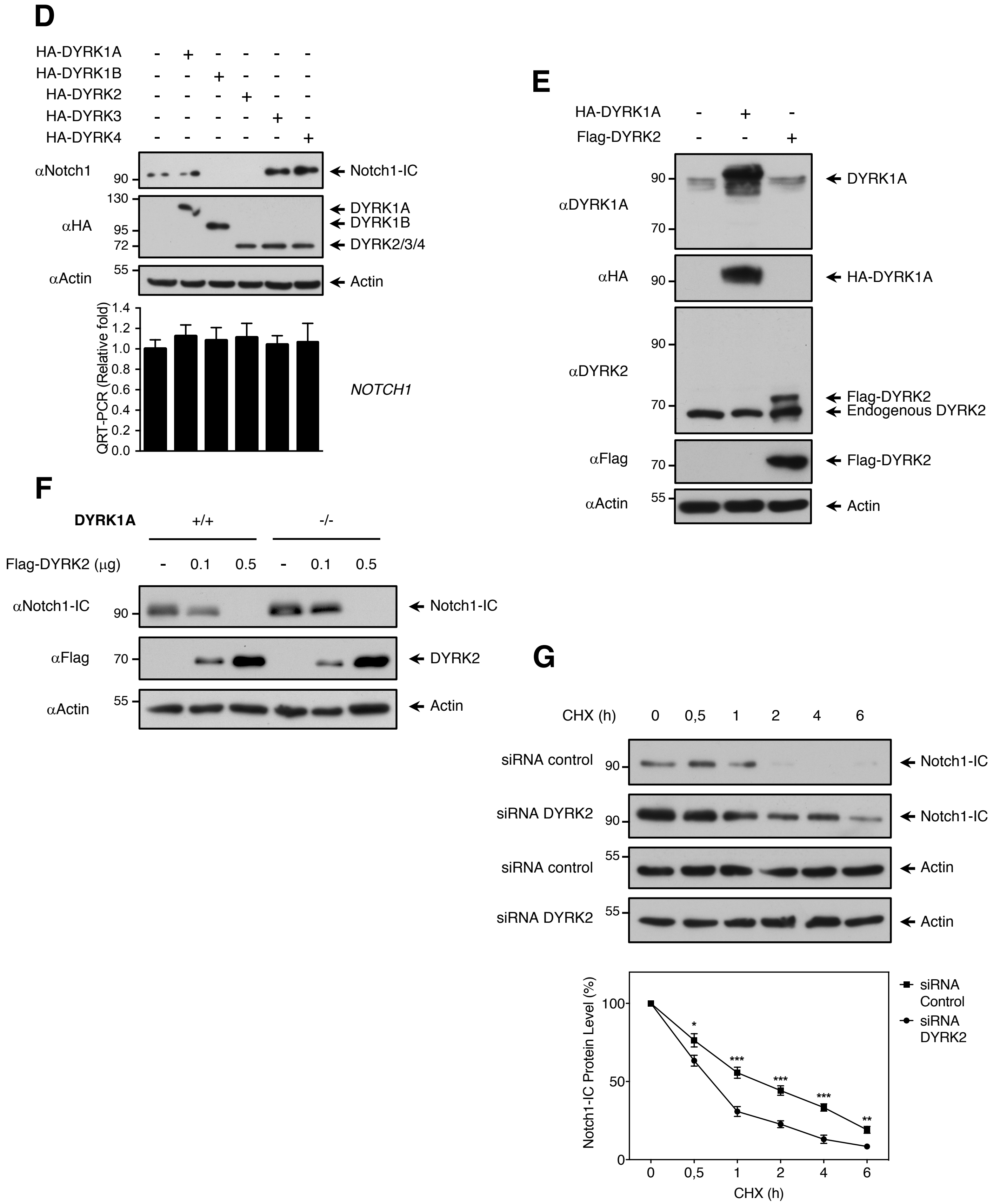
D
HA-DYRK1A
HA-DYRK1B
HA-DYRK2
HA-DYRK3
HA-DYRK4
αNotch1
αHA
αActin
90
130
95
72
55
Notch1-IC
DYRK1A
DYRK1B
DYRK2/3/4
Actin
QRT-PCR (Relative fold)
1.4
1.2
1.0
0.8
0.6
0.4
0.2
0.0
NOTCH1
E
HA-DYRK1A
Flag-DYRK2
αDYRK1A
αHA
αDYRK2
αFlag
αActin
90
70
55
DYRK1A
HA-DYRK1A
Flag-DYRK2
Endogenous DYRK2
Actin
F
DYRK1A
+/+
-/-
Flag-DYRK2 (μg)
0.1
0.5
αNotch1-IC
αFlag
αActin
Notch1-IC
DYRK2
Actin
G
CHX (h)
0
0,5
1
2
4
6
siRNA control
siRNA DYRK2
Notch1-IC
Actin
Notch1-IC Protein Level (%)
100
50
0
siRNA Control
siRNA DYRK2
*
***
**

Fig. S1

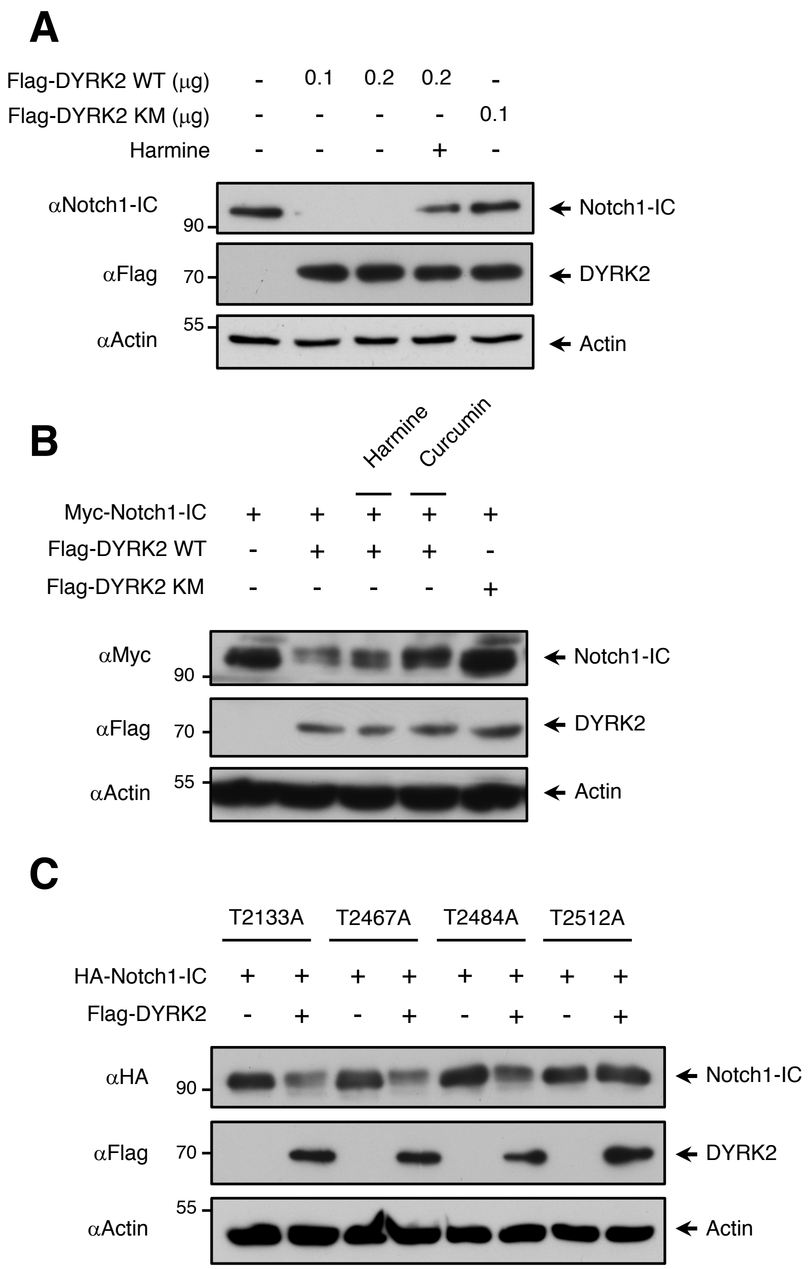
A
Flag-DYRK2 WT (μg) - 0.1 0.2 0.2 -
Flag-DYRK2 KM (μg) - - - - 0.1
Harmine - - - + -
αNotch1-IC
90
Notch1-IC
αFlag
70
DYRK2
55
αActin
Actin
B
Harmine
Curcumin
Myc-Notch1-IC + + + + +
Flag-DYRK2 WT - + + + -
Flag-DYRK2 KM - - - - +
αMyc
90
Notch1-IC
αFlag
70
DYRK2
55
αActin
Actin
C
T2133A
T2467A
T2484A
T2512A
HA-Notch1-IC + + + + + + + +
Flag-DYRK2 - + - + - + - +
αHA
90
Notch1-IC
αFlag
70
DYRK2
55
αActin
Actin

Fig. S2

**A**

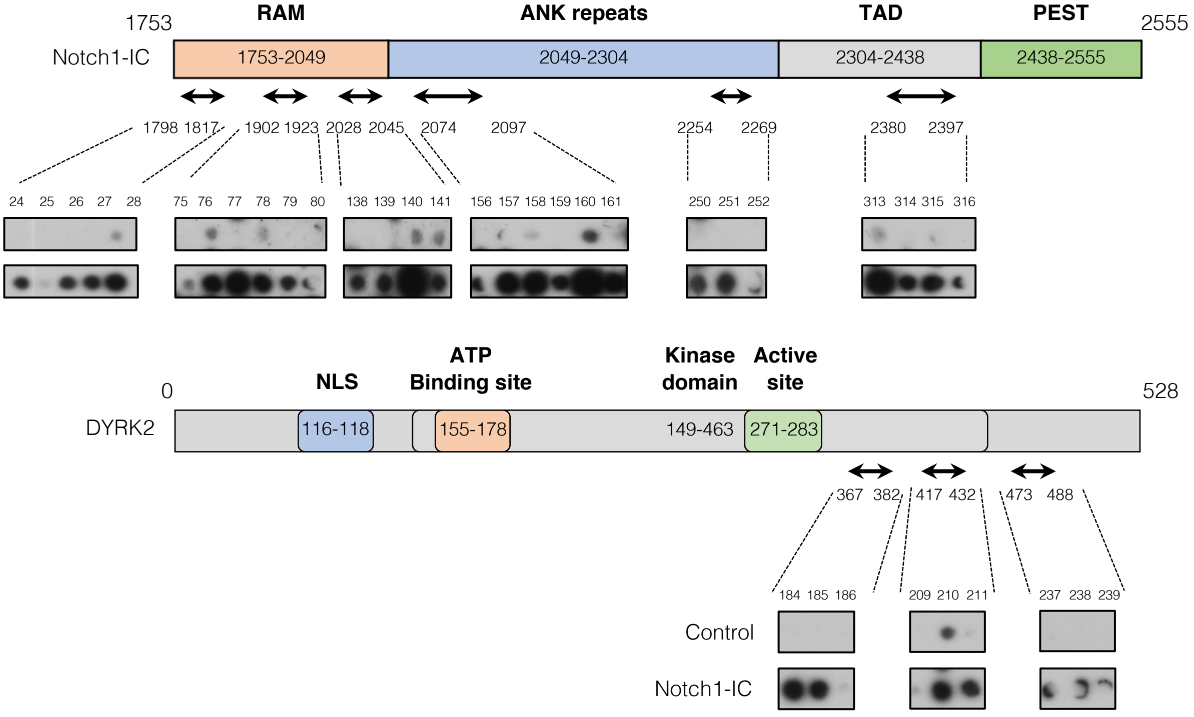


**B**

**Notch-IC**

| | |
|---|---|
| 24 | KNASDGALMDDN |
| 25 | ASDGALMDDNQN |
| 26 | DGALMDDNQNEW |
| 27 | ALMDDNQNEWGD |
| 28 | MDDNQNEWGDED |
| 75 | EEEDAPAVISDF |
| 76 | EDAPAVISDFIY |
| 77 | APAVISDFIYQG |
| 78 | AVISDFIYQGAS |
| 79 | ISDFIYQGASLH |
| 80 | DFIYQGASLHNQ |
| 138 | GKSALHWAAAVN |
| 139 | SALHWAAAVNNV |
| 140 | LHWAAAVNNVDA |
| 141 | WAAAVNNVDAAV |
| 156 | YETAKVLLDHFA |
| 157 | TAKVLLDHFANR |
| 158 | KVLLDHFANRDI |
| 159 | LLDHFANRDITD |
| 160 | DHFANRDITDHM |
| 161 | FANRDITDHMDR |
| 162 | NRDITDHMDRLP |
| 250 | EMAALGGGGRLA |
| 251 | AALGGGGRLAFE |
| 252 | LGGGGRLAFETG |
| 313 | VQTQQVQPQNLQ |
| 314 | TQQVQPQNLQMQ |
| 315 | QVQPQNLQMQQQ |
| 316 | QPQNLQMQQQNL |

**DYRK2**

| | |
|---|---|
| 184 | MPSQKLLDASKR |
| 185 | SQKLLDASKRAK |
| 186 | KLLDASKRAKNF |
| 209 | PPESREWGNALK |
| 210 | ESREWGNALKGC |
| 211 | REWGNALKGCDD |
| 237 | EKTSVKRITEST |
| 238 | TSVKRITESTGA |
| 239 | VKRITESTGAIT |

Fig. S4

**C**

**HA-Notch1-RAM1:**

1798 … 1817

KNASDGALMDDNQNEWGDED WT
KNASDGAL**AAA**NQNEWGDED Mutant

**HA-Notch1-RAM2:**

1902 … 1923

EEEDAPAVISDFIYQGASLHNQ WT
EEEDAPAVI**AAA**IYQGASLHNQ Mutant

**HA-Notch1-RAM3:**

2028 … 2045

GKSALHWAAAVNNVDAAV WT
GKSALHWAA**KEA**NVDAAV Mutant

**HA-Notch1-ANK1:**

2074 … 2097

YETAKVLLDHFANRDITDHMDRLP WT
YETAKVLL**AAAA**NRDITDHMDRLP Mutant

**HA-Notch1-ANK2:**

2254 … 2269

EMAALGGGGRLAFETG WT
EMAALG**AAA**RLAFETG Mutant

**HA-Notch1-TAD1:**

2380 … 2397

VQTQQVQPQNLQMQQQNL WT
VQTQQVQ**AAA**LQMQQQNL Mutant

**D**

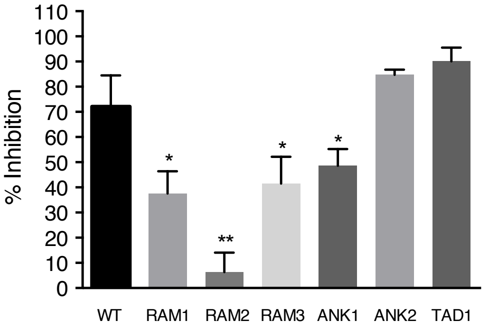


Fig. S4

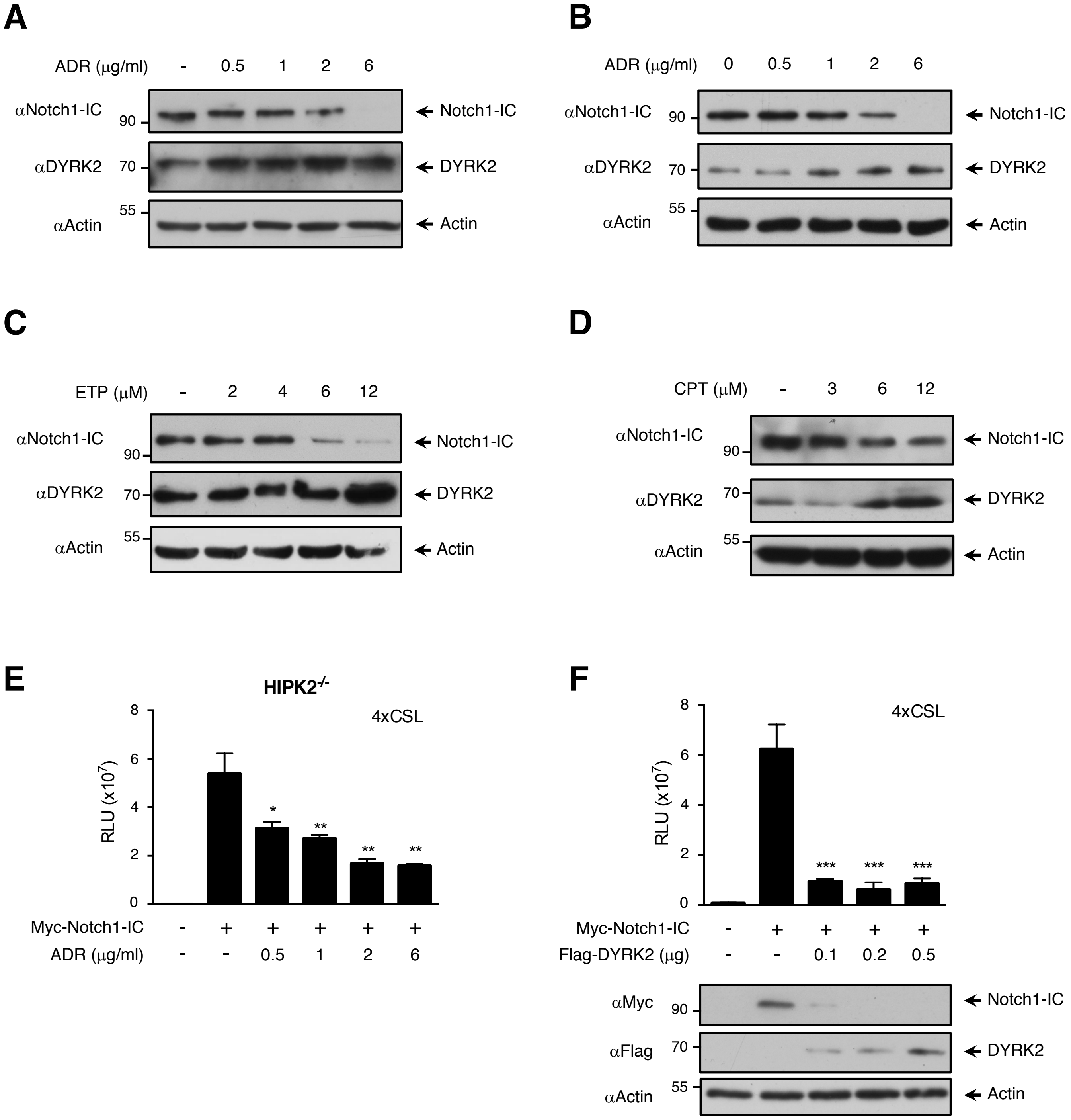

A
ADR (μg/ml) - 0.5 1 2 6
αNotch1-IC 90
αDYRK2 70
55
αActin
← Notch1-IC
← DYRK2
← Actin
B
ADR (μg/ml) 0 0.5 1 2 6
αNotch1-IC 90
αDYRK2 70
55
αActin
← Notch1-IC
← DYRK2
← Actin
C
ETP (μM) - 2 4 6 12
αNotch1-IC 90
αDYRK2 70
55
αActin
← Notch1-IC
← DYRK2
← Actin
D
CPT (μM) - 3 6 12
αNotch1-IC 90
αDYRK2 70
55
αActin
← Notch1-IC
← DYRK2
← Actin
E
HIPK2-/-
4xCSL
RLU (x10^7)
Myc-Notch1-IC - + + + + +
ADR (μg/ml) - - 0.5 1 2 6
F
4xCSL
RLU (x10^7)
Myc-Notch1-IC - + + + +
Flag-DYRK2 (μg) - - 0.1 0.2 0.5
αMyc 90
αFlag 70
55
αActin
← Notch1-IC
← DYRK2
← Actin


Fig. S5

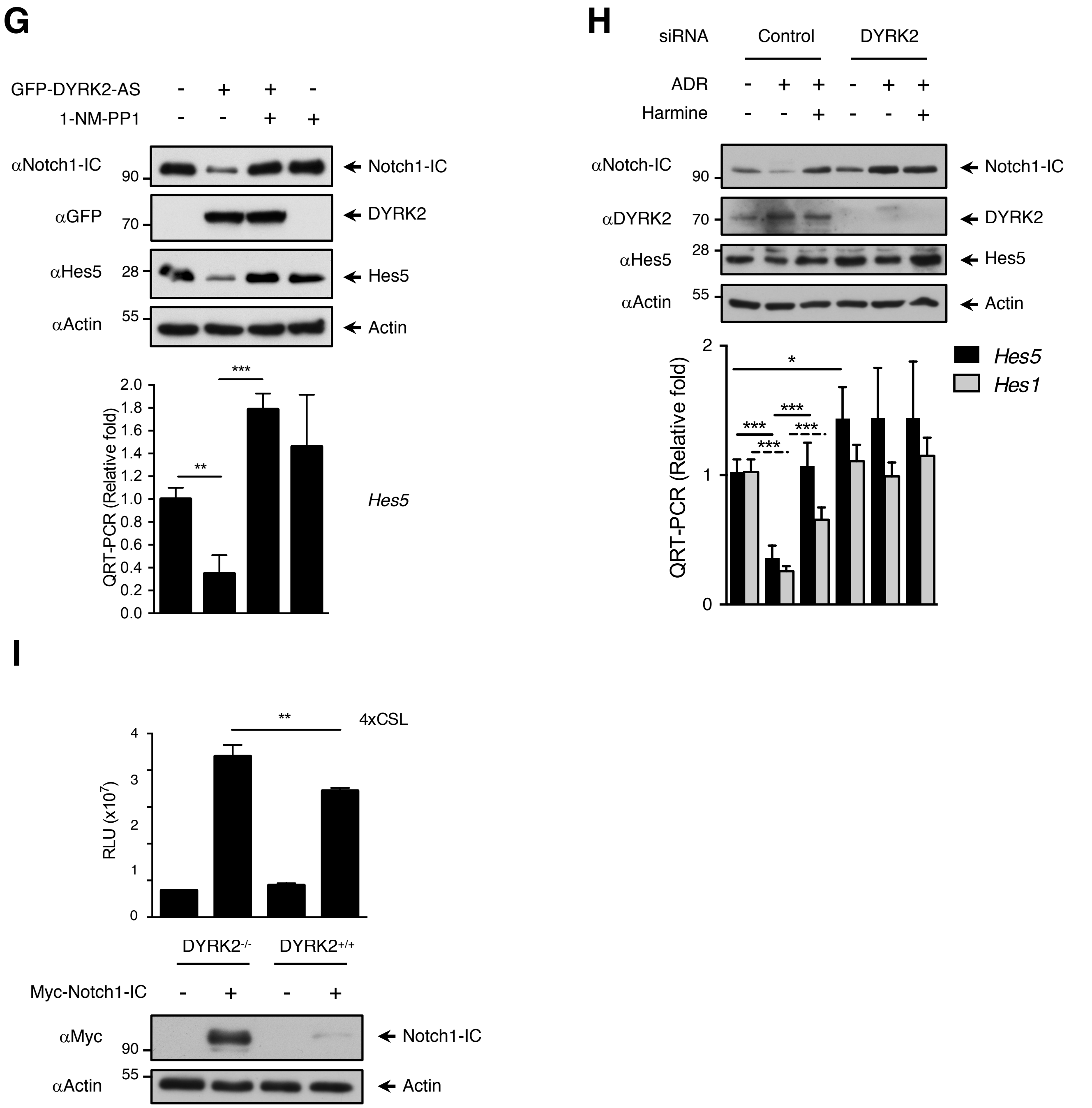
G
GFP-DYRK2-AS - + + -
1-NM-PP1 - - + +
αNotch1-IC 90 Notch1-IC
αGFP 70 DYRK2
αHes5 28 Hes5
αActin 55 Actin
QRT-PCR (Relative fold)
Hes5
**
***
H
siRNA Control DYRK2
ADR - + + - + +
Harmine - - + - - +
αNotch-IC 90 Notch1-IC
αDYRK2 70 DYRK2
αHes5 28 Hes5
αActin 55 Actin
QRT-PCR (Relative fold)
Hes5
Hes1
I
4xCSL
RLU (x10^7)
DYRK2-/- DYRK2+/+
Myc-Notch1-IC - + - +
αMyc 90 Notch1-IC
αActin 55 Actin

Fig. S5

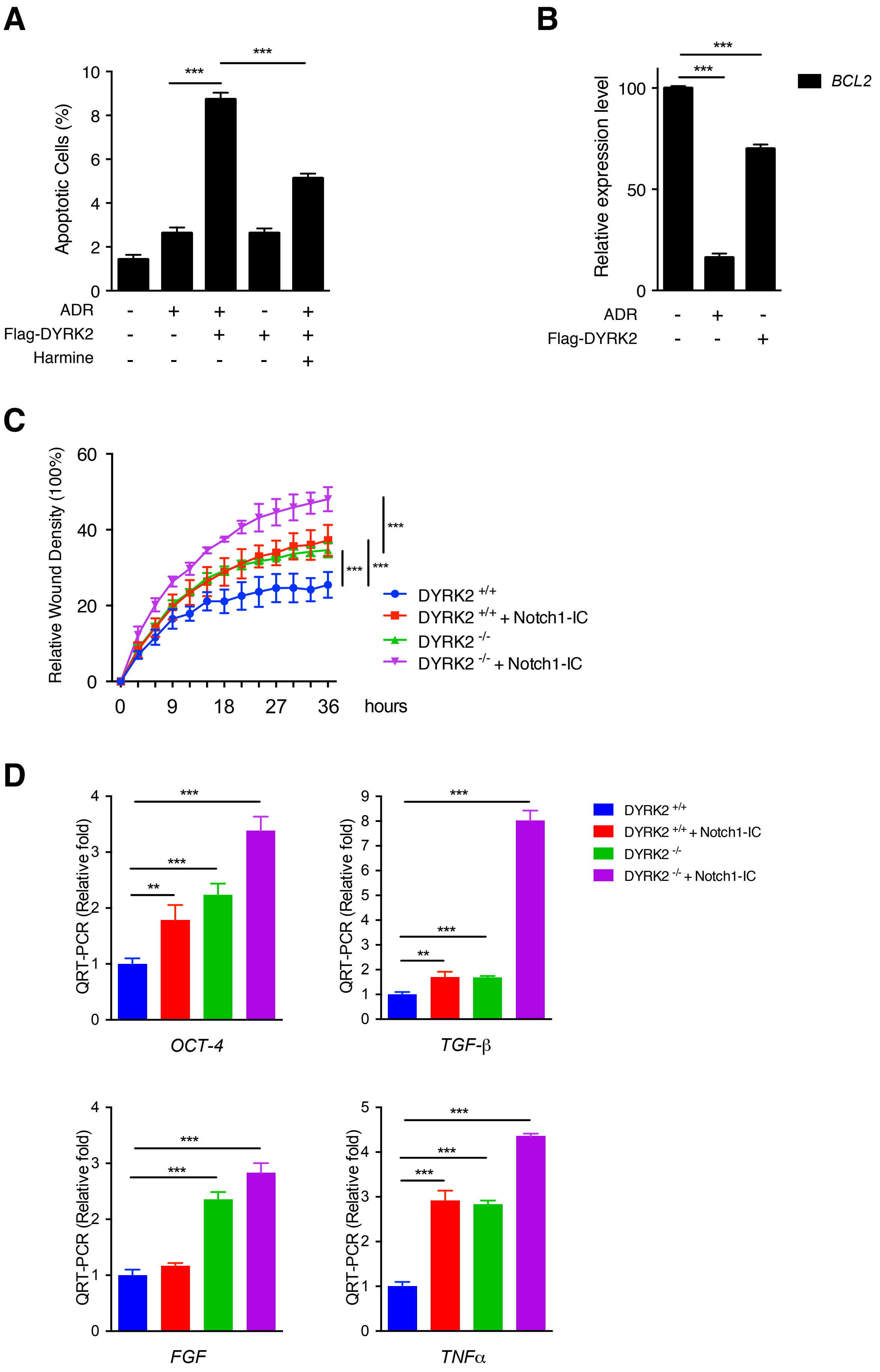
A
Apoptotic Cells (%)
ADR
Flag-DYRK2
Harmine
B
Relative expression level
BCL2
C
Relative Wound Density (100%)
hours
DYRK2 +/+
DYRK2 +/+ + Notch1-IC
DYRK2 -/-
DYRK2 -/- + Notch1-IC
D
QRT-PCR (Relative fold)
OCT-4
TGF-β
FGF
TNFα

Fig. S6
